# Field assessment of yield and its contributing traits in cowpea treated with lower, intermediate, and higher doses of gamma rays and sodium azide

**DOI:** 10.3389/fpls.2023.1188077

**Published:** 2023-07-14

**Authors:** Aamir Raina, Samiullah Khan

**Affiliations:** ^1^ Mutation Breeding Laboratory, Department of Botany, Aligarh Muslim University, Aligarh, India; ^2^ Botany Section, Women’s College, Aligarh Muslim University, Aligarh, India

**Keywords:** bio-physiological traits, quantitative traits, correlation analysis, path analysis, cluster analysis

## Abstract

Across the globe, plant breeders of different organizations are working in collaboration to bring preferred traits to crops of economic importance. Among the traits, “high yielding potential” is the most important as it is directly associated with food security and nutrition, one of the sustainable development goals. The Food and Agriculture Organization acknowledges plant breeders’ role and efforts in achieving local and global food security and nutrition. Recognizing the importance of pulses and increasing pressure on food security, the United Nations General Assembly declared 2016 the “International year of Pulses” owing to their preferred traits such as climate change resilience, wide adaptability, low agriculture input, and protein- and nutrient-rich crops. Keeping all these developments in consideration, we initiated an induced mutagenesis program by treating cowpea (*Vigna unguiculata* L. Walp.) with different doses of gamma rays and sodium azide aiming to enhance the yielding potential of an otherwise outstanding variety viz., Gomati VU-89 and Pusa-578. We noticed a substantial increase in mean values of agronomic traits in putative mutants raised from seeds treated with lower and intermediate doses of mutagens. Statistical analysis such as correlation, path, hierarchical clustering analysis (HCA), and principal component analysis (PCA) were used to assess the difference between mutagenized and control populations. A significant and positive correlation of yield with yield-attributing traits was recorded. However, among all the yield attributing traits, seeds per pod (SPP) depicted the maximum direct impact upon yield, and therefore, working on this trait may yield better results. A widely used PCA revealed 40.46% and 33.47% of the total variation for var. Gomati VU-89 and var. Pusa-578, respectively. Cluster analysis clustered treated and control populations into separate clusters with variable cluster sizes. Cluster V in the variety Gomati VU-89 and cluster V and VI in the variety Pusa 578 comprised of putative mutants were higher yielding and hence could be recommended for selection in future breeding programs. We expect to release such mutant lines for farmer cultivation in Northern parts of India depending on the performance of such high-yielding mutant lines at multilocations.

## Introduction

**Figure 1:**
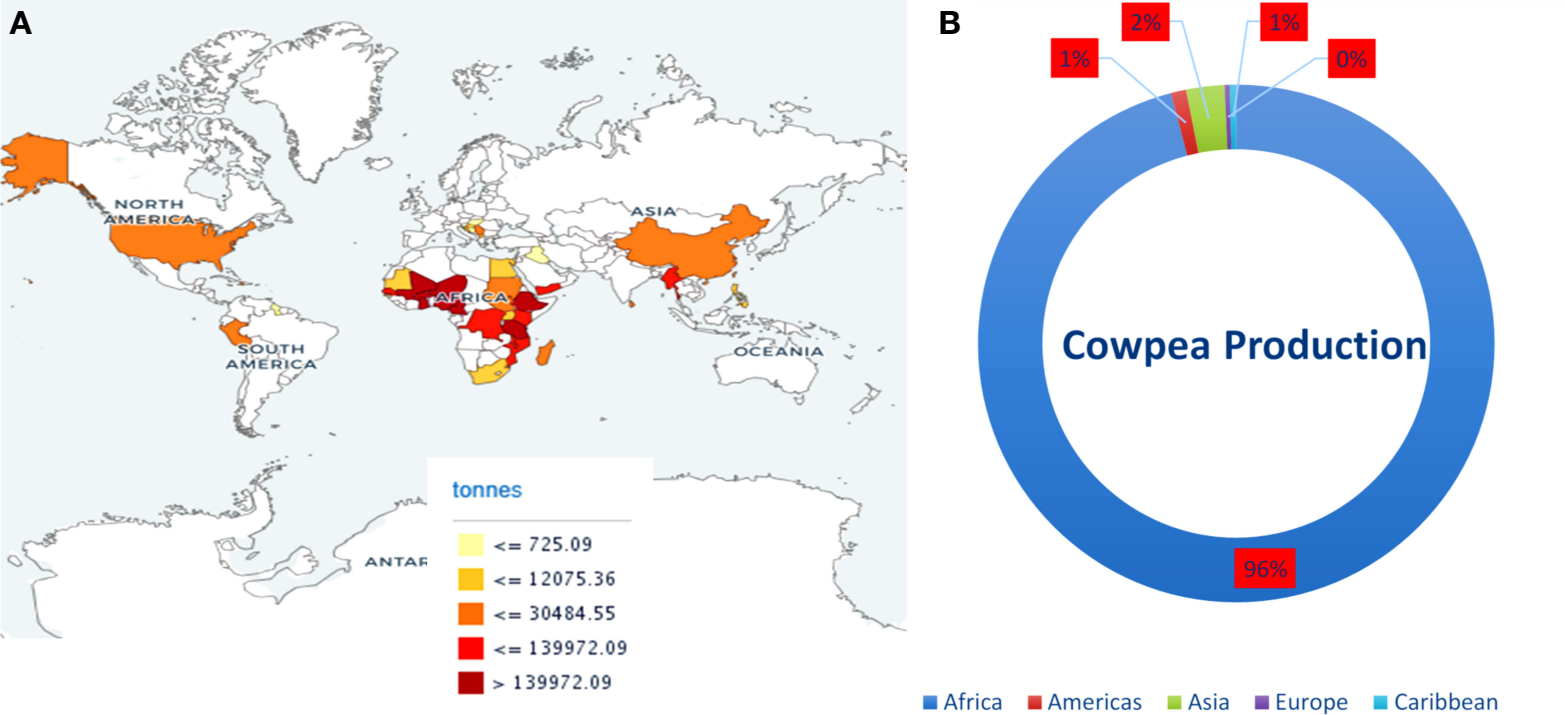
**(A)** Map showing cowpea producing countries and their production. **(B)** Average production of cowpea (dry) in continents from 2011-2021. Source: FAOSTAT https://www.fao.org/faostat (accessed on 20 June, 2023).

Cowpea, a self-fertilizing diploid with chromosome number 2n = 2x = 22, genome size 620 Mb, belongs to the Fabaceae family is a warm-season grain legume widely cultivated across a wide range of dry ecologies (Arumuganathan and Earle, 1991; Lo et al., 2020). Cowpea is an inexpensive plant-based protein resource, ranks second in diet value after cereals, and complements a protein-deficient cereal-based human diet. It is cultivated for its tender green leaves, unripe pods, and dry grains as a major source of dietary protein for millions of people across the globe (Uzogara and Ofuya, 1992). Its grains are rich in protein, carbohydrates, dietary fiber, vitamins, antioxidants, polyphenols, and polyunsaturated fatty acids (Ngoma et al., 2018; Ketema et al., 2020; Nkomo et al., 2021). Cowpeas are vital constituents of sustainable agriculture and are critical in enhancing human and livestock health. It offers vital ecological services through its capability to biologically fix nitrogen, recycle nutrients, improve soil carbon content, and diversify cropping systems (Nkomo et al., 2021). Cowpea is often referred to as climate-smart crop due to its dual resilience against heat and drought stress, low water footprint and agriculture input, and ability to thrive under diverse and adverse environmental conditions where other legumes cannot grow. Globally, 14.4 million hectares (ha) of land are devoted to its cultivation, and more than 8.9 million tonnes are produced annually. Africa contributes the maximum annual cowpea produce among the continents, followed by Asia and America (**Figure 1**). The main cowpea-producing countries are Nigeria, Niger, and Burkina Faso; however, the annual mean cowpea yield in Niger and Burkina Faso was 4,169 and 4,818 hectogram/hectare (hg/ha), respectively, which is less as compared to the world average of 6,163 hg/ha (FAOSTAT accessed on 06 Aug 2021). The unavailability of high-yielding cowpea varieties is the main obstacle to achieving higher production (Wamalwa et al., 2016; Raina et al., 2020). Therefore, cowpea breeders and geneticists have employed traditional and modern breeding strategies to improve yield, grain quality, and nutritional and nutraceutical properties. However, traditional breeding strategies are arduous, time-consuming, and inefficient in achieving the desired goals.

Among the breeding strategies, induced mutagenesis is a coherent tool for improving yield and yield-attributing traits. With wide public acceptance and no regulatory restrictions unlike transgenic plants, the mutation breeding approach has been most successful in developing thousands of elite mutant varieties in hundreds of plant species. Induced mutagenesis has successfully developed 3402 mutant varieties (https://mvd.iaea.org/ accessed on 08 July 2023). However, cowpea is the neglected crop in induced mutagenesis, and only 16 cowpea mutant varieties have been developed so far. Hence, it is imperative to develop mutants with a preferred set of traits through induced mutagenesis. Various cultivars were treated with a wide range of gamma (γ) ray doses in developing these cowpea mutant varieties. No information on the optimum doses of γ rays that could be used for the yield improvement of cowpea is available. Similarly, none of the workers have been successful in developing cowpea mutant varieties using optimum doses of sodium azide (SA). SA can induce AT→GC base pair transition and transversion (**Supplementary Figure 1**). Considering the need for optimization of γ rays and SA doses, an experimental study was undertaken by treating two cowpea varieties with single and combined doses of γ rays and SA. The γ rays are ionizing radiations with a short, high-energy wavelength and can interact and alter DNA base pairs. The γ rays cause hydrolysis of water that leads to the production of reactive free radicals that disturb DNA–DNA cross-links and eventually result in mutations (Reisz et al., 2014).

The yield is a complex trait governed by multiple genes that impart a small cumulative effect. In grain legumes, yield is total performance and the association of yield-contributing traits. The association between yield-contributing traits is achieved by assessing the correlation coefficients. Therefore, these traits are important and getting higher mean values is the ultimate aim of the breeding program. Even the success of the breeding program relies on the correlation direction and degree among yield-attributing traits (Ahmad and Saleem, 2003). This is why correlation analysis is important in formulating the breeding design and selection pressure (Sarawgi et al., 1997). Correlation analysis evaluates the degree and direction of the association between traits, enabling the breeders to exercise indirect selection much faster than traditional direct selection on the desired trait (Cruz et al., 2012).

Even though correlation analysis is widely used in analyzing the association between traits, it does not count the direct and indirect effects of these traits and provides zero information on cause and effect (Santos et al., 2014). Therefore, it can lead to errors in the selection strategy, and it not enough to assess the relationship between traits without including the direct and indirect effects. Hence, a breeder must have extensive knowledge of the direct and indirect relationship among yield-contributing traits in the selection of plants for breeding. To have an accurate picture of the association among traits, Wright (1921) formulated path analysis that quantifies the direct and indirect impact of the predictor variable upon its response variable. It also reveals the comparative significance of each trait contributing to the yield (Li, 1975; Cruz and Regazzi, 2006). It helps in the selection procedure and equips the breeders to isolate mutants simultaneously based on two or more traits (Salahuddin et al., 2010). Path analysis enables breeders to visualize direct and indirect effects, and hence, it has been widely used in crops such as *Passiflora edulis* (Araújo et al., 2007), *Brassica napus* (Coimbra et al., 2005), *Triticum aestivum* (Carvalho et al., 1999), *Theobroma cacao* (Almeida et al., 1994), and *Glycine max* (Bizeti et al., 2004). The overall aims of correlation and path analysis are to assess the role of yield-contributing traits in the determination of yield and to design selection criteria to isolate mutants with desirable traits for breeding programs.

## Materials and methods

### Plant material

The seeds of cowpea varieties viz., Gomati VU-89 and Pusa-578, were obtained from the National Bureau of Plant Genetic Resources and were irradiated at National Botanical Research Institute, Lucknow, India, with 100, 200, 300, 400, 500, 600, 700, 800, 900, and 1,000 Gy doses of γ rays at a dose rate of 11.58 Gy/min. For chemical treatments, seeds were presoaked in double-distilled water for 6 h and then treated with several doses of SA such as 0.01%, 0.02%, 0.03%, 0.04%, 0.05%, 0.06%, 0.07%, 0.08%, 0.09%, and 0.1% of SA for 9 h at Mutation Breeding Laboratory, Botany Department, Aligarh Muslim University (AMU), Aligarh, India. For combined mutagen treatments, seeds were treated with 100 Gy γ rays + 0.01% SA, 200 Gy γ rays + 0.02% SA, 300 Gy γ rays + 0.03% SA and 400 Gy γ rays + 0.04% SA, 500 Gy γ rays + 0.05% SA, 600 Gy γ rays + 0.06% SA, 700 Gy γ rays + 0.07% SA, 800 Gy γ rays + 0.08% SA, 900 Gy γ rays + 0.09% SA, and 1,000 Gy γ rays + 0.1% SA. We found that doses beyond 400 Gy γ rays, 0.04% SA, and 400 Gy γ rays + 0.04% SA are detrimental and caused more than 50% reduction in seed germination and hence were discarded.

### Field assessment

During mid-April 2014, 300 seeds/treatment/variety totaling 7,800 were sown in the agriculture field, AMU, Aligarh, in a randomized complete block design keeping 0.3-m seed-to-seed and 0.6-m row-to-row distance. The seeds were sown in 10 replications of 30 seeds. Each block (1.8 × 3 m) consisted of one replication from each treatment in the field (23.5 × 40 m) (**Supplementary Figure 2**). All the seeds from M_1_ plants were harvested separately during mid-October 2014 and stored for raising subsequent generations.

### Nitrate reductase activity

Secondary leaflets of M_1_ seedlings were collected and washed thoroughly two to three times with running water after 10–15 days of sowing (DAS) to estimate nitrate reductase activities following the protocol of Jaworski (1971). The detailed methodology is given separately in **Supplementary Files**.

### Chlorophyll and carotenoid contents

For estimation of chlorophyll and carotenoid contents, secondary leaflets of M_1_ seedlings were collected and washed thoroughly two to three times with running water after 10–15 days after sowing (DAS) following the protocol of MacKinney (1941). The detailed methodology is given separately in **Supplementary Files**. The pigment contents were measured on a fresh weight basis (mg.g^-1^FW) following the formula of Arnon (1949):


$$\text{ Total chlorophyll }=\;\left\{20.2\left({\text{OD}}_{645}\right)\;+\;8.02\left({\text{OD}}_{663}\right)\right\}\;\times \;\frac{\text{V}}{1,000\text{ x W}}$$

$$\text{Carotenoids}=\;\frac{7.6\;\left({\text{OD}}_{480}\right)-\;1.49\;\left({\text{OD}}_{510}\right)}{\text{d }\times \;1,000\;\times \text{ W}}\times \text{ V}$$

where OD_645_, OD_663_, OD_480_, OD_510_ = optical densities at OD_645_, OD_663_, OD_480_, and OD_510_, respectively.

V= Volume of an extract
W = Mass of leaf tissues
d = Length of light path (d = 1.4 cm).

### Quantitative traits

We chose 30 random mutagen dose-treated and untreated plants to generate the mean data of 10 quantitative traits. For plant height (PH), we measured the total length of plants from base to the apex. We recorded the days to flowering (DF), starting from seed sowing to the appearance of first floral buds. Similarly, total growth period or days to maturity (DM) was recorded by counting days from seed sowing to harvest. In cowpea, flowers develop at different times, which lead to a multiple pod set, and hence, pods were harvested three to four times manually from September to October 2014. For pods per plant (PPP), we counted a total number of pods collected in a sequential harvests. Similarly, we randomly chose 60 pods from 30 plants to calculate the mean number of seeds per pod (SPP). The mean number of branches per plant was recorded by counting the branches during the flowering period. Mean seed weight (SW) was measured by taking the weight of 100 seeds. Pod length (PL) was recorded by measuring the length from the base to the pod tip. Plant yield (PY) was measured by taking the weight of total seeds harvested from 30 randomly selected plants. The harvest index (HI) was measured as a ratio of seed yield to biological yield or dry weight of aboveground parts subjected to 7 days of continuous sun drying.

### Statistical analysis

To visualize the significance (P ≤ 0.05) of the data (number of replications, n = 30), one-way analysis of variance and Duncan’s multiple range test was conducted with the help of R software (R Core Team, 2019). Correlation and path coefficients were calculated from replicated data of 30 plants in control and mutagenized populations using SAS and IBM SPSS AMOS 26 software, respectively. Hierarchical Cluster Analysis (HCA) allowed us to visualize the heterogeneity produced among different mutagenized populations. The results were presented graphically in the form of dendrograms using SPSS version 16.0 (Team EQX). The biplot PCA graphs were constructed by taking the mean data of quantitative traits from control and treated populations using Past software version 3.26 (Hammer et al., 2001).

## Results

### Biophysiological traits

**Figure 2:**
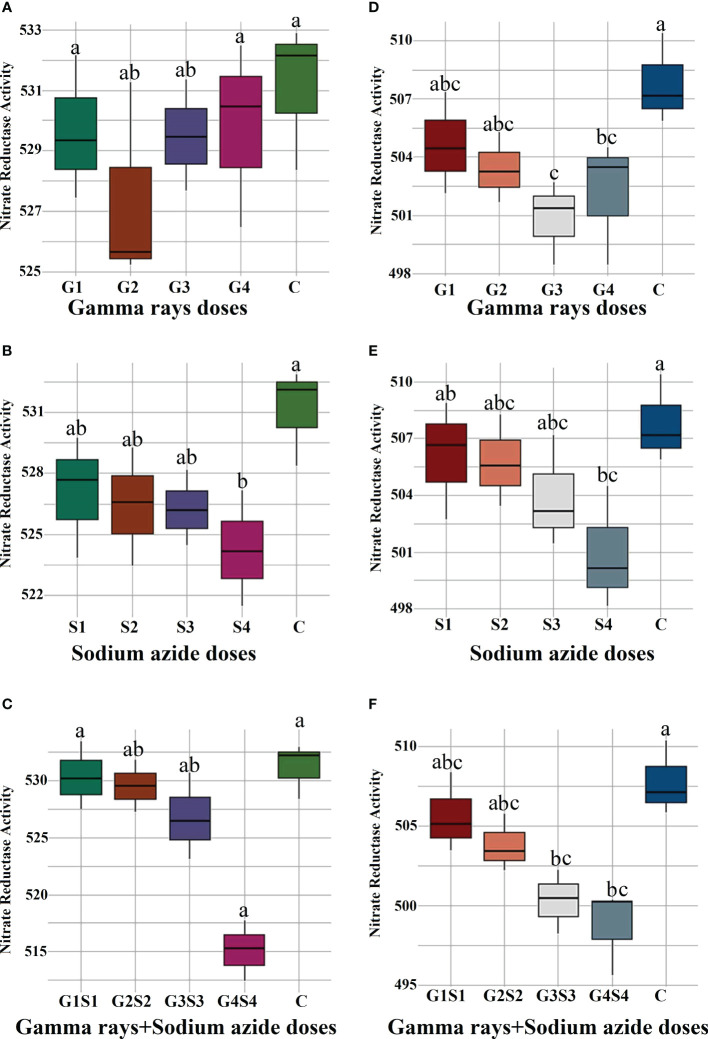
Effects of different doses of γ rays, SA, and γ rays + SA on nitrate reductase activity **(A–C)** in var. Gomati VU-89 and **(D–F)** in var. Pusa-578. The data are presented as mean and standard error (n = 30). #Means followed by the same letter is not different at 5% level of significance, based on the DMRT. C, Control; G1, 100 Gy γ rays; G2, 200 Gy γ rays; G3, 300 Gy γ rays; G4, 400 Gy γ rays; S1, 0.01% SA; S2, 0.02% SA; S3, 0.03% SA; S4, 0.04% SA; G1 +S1, 100 Gy γ rays+0.01% SA; G2+S2, 200 Gy γ rays+0.02% SA; G3+S3, 300 Gy γ rays+0.03% SA; G4+S4, 400 Gy γ rays+0.04% SA.

**Figure 3:**
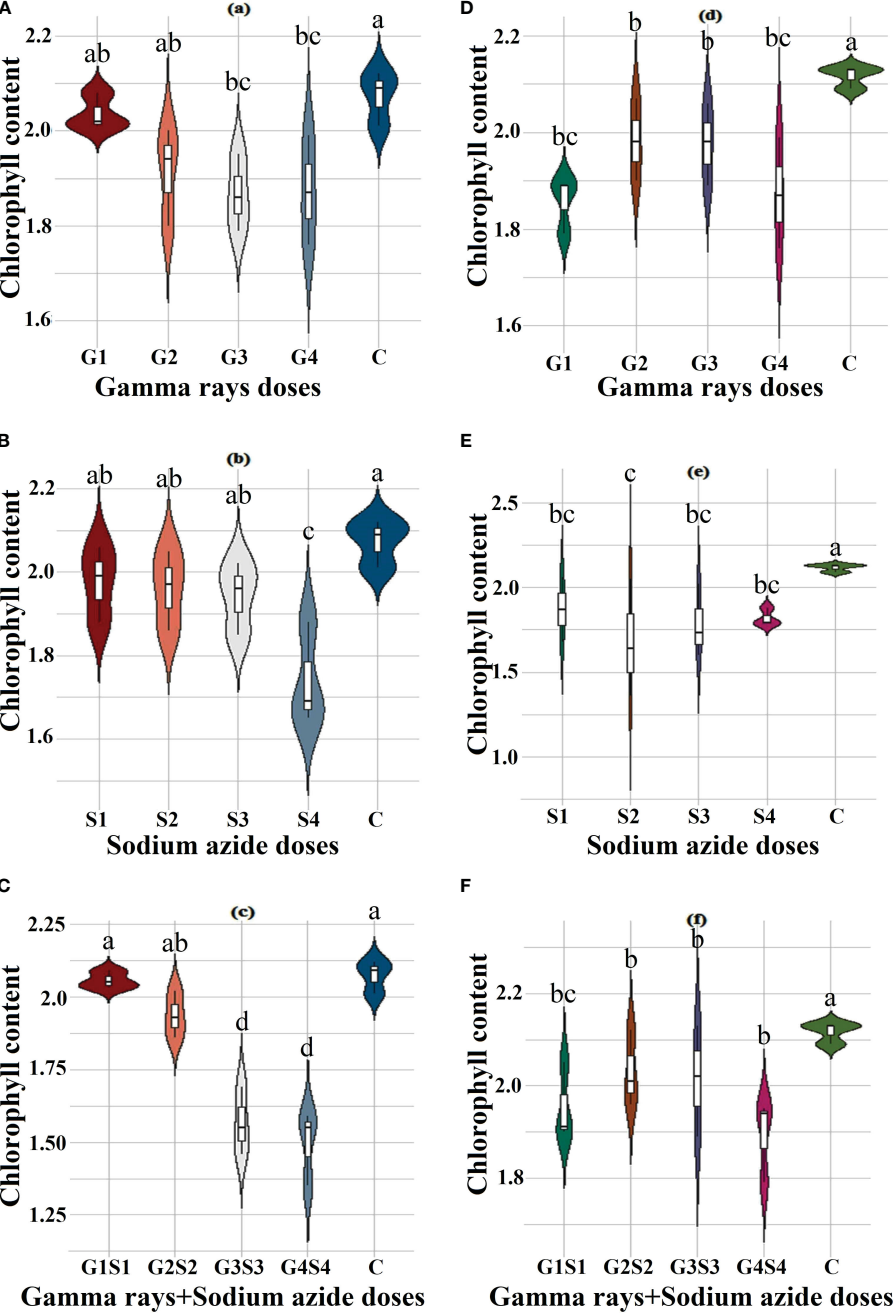
Effects of different doses of γ rays, SA, and γ rays + SA on chlorophyll contents **(A–C)** in var. Gomati VU-89 and **(D–F)** in var. Pusa-578. The data are presented as mean and standard error (n = 30). #Means followed by the same letter is not different at 5% level of significance, based on the DMRT. C, Control; G1, 100 Gy γ rays; G2, 200 Gy γ rays; G3, 300 Gy γ rays; G4, 400 Gy γ rays; S1, 0.01% SA; S2, 0.02% SA; S3, 0.03% SA; S4, 0.04% SA; G1 +S1, 100 Gy γ rays+0.01% SA; G2+S2, 200 Gy γ rays+0.02% SA; G3+S3, 300 Gy γ rays+0.03% SA; G4+S4, 400 Gy γ rays+0.04% SA.

**Figure 4:**
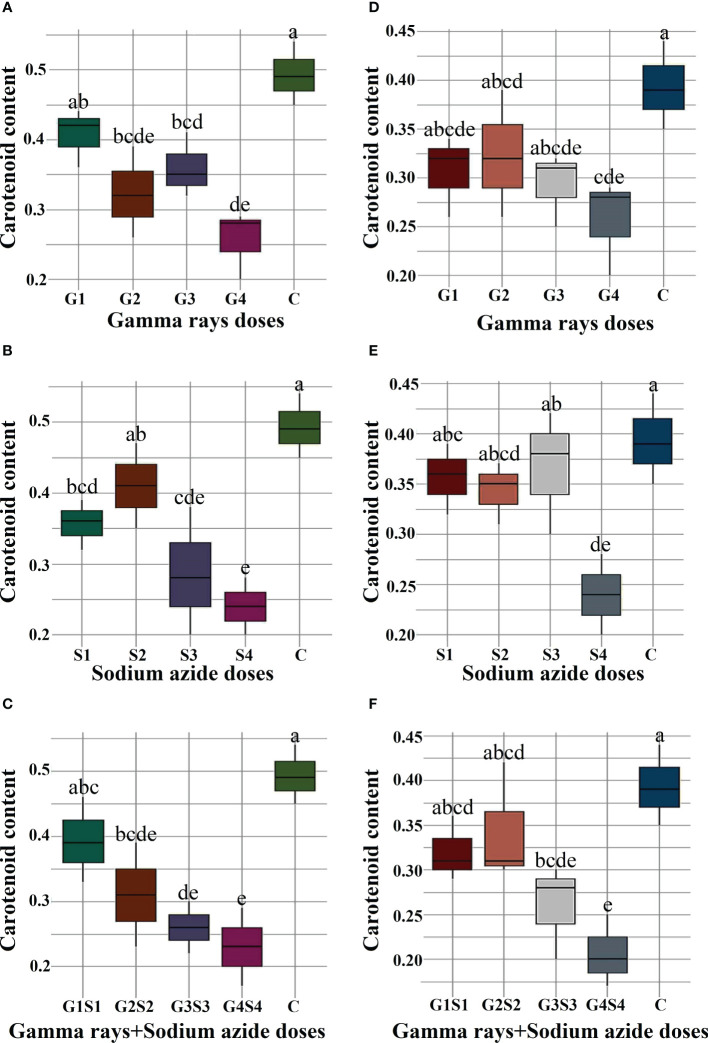
Effects of different doses of gamma (γ) rays, SA, γ rays + SA on carotenoid contents **(A–C)** in var. Gomati VU-89 and **(D–F)** in var. Pusa-578. The data are presented as mean and standard error (n = 30). #Means followed by the same letter is not different at 5% level of significance, based on the Duncan's Multiple Range Test (DMRT). C, Control; G1, 100 Gy γ rays; G2, 200 Gy γ rays; G3, 300 Gy γ rays; G4, 400 Gy γ rays; S1, 0.01% SA; S2, 0.02% SA; S3, 0.03% SA; S4, 0.04% SA; G1 +S1, 100 Gy γ rays+0.01% SA; G2+S2, 200 Gy γ rays+0.02% SA; G3+S3, 300 Gy γ rays+0.03% SA; G4+S4, 400 Gy γ rays+0.04% SA.

The results revealed a random and dose-independent decline in the biophysiological traits in the treated population of both varieties (**Figures 2**–**4**). A significantly higher Nitrate Reductase Activity (NRA) (531.12; 507.79 nmol.h^-1^.g^-1^FW), chlorophyll (2.07; 2.42 mg.g^-1^FW), and carotenoid contents (0.49; 0.39 mg. g^-1^FW) were recorded in a control population of the varieties Gomati VU-89 and Pusa-578, respectively. The combined mutagen doses induced a drastic reduction in biophysiological traits.

### Quantitative traits

The results revealed that 200 Gy γ rays, 300 Gy γ rays, 400 Gy γ rays, 0.04% SA, 200 Gy γ rays + 0.02% SA, 300 Gy γ rays + 0.03% SA, and 400 Gy γ rays + 0.04% SA treatments induced a statistically significant decrease in mean PH in the var. Gomati VU-89 (**Table 1**). In the var. Pusa-578, all the mutagen doses except 400 Gy γ rays, 300 Gy γ rays + 0.03% SA, and 400 Gy γ rays + 0.04% SA treatments induced a statistically insignificant decrease in mean PH (**Table 2**). The highest reduction in mean PH was noted in 400 Gy γ rays + 0.04% SA treatment in Gomati VU-89 (174.13 cm) and Pusa-578 (173.05 cm). All the mutagen doses except 200 Gy γ rays, 300 Gy γ rays, and 100 Gy γ rays + 0.01% SA in the var. Gomati VU-89 and 100 Gy γ rays, 200 Gy γ rays, 300 Gy γ rays, 0.03% SA, and 0.04% SA in the var. Pusa-578 induced a decrease in the mean number of DF (**Table 1**). The highest reduction in the mean number of DF was recorded in the 400 Gy γ rays + 0.04% SA treatment in the var. Gomati VU-89 (77.93) and 0.02% SA and 300 Gy γ rays + 0.03% SA treatments in the var. Pusa-578 (86.43). The results revealed all the mutagen doses except 0.02% SA, 100 Gy + 0.01% SA in the var. Gomati VU-89 and 100 Gy, 0.01% SA, 0.02% SA, 100 Gy + 0.01% SA, and 200 Gy + 0.02% SA in the var. Pusa-578 induced a statistically insignificant decrease in the mean number of DM in both varieties (**Tables 1**, **2**). The highest decrease in the mean number of DM was noted in 400 Gy γ rays + 0.04% SA treatment in the varieties Gomati VU-89 (148.83) and Pusa-578 (154.80). The results revealed 200 Gy γ rays, 0.02% SA, 100 Gy γ rays + 0.01% SA, 200 Gy γ rays + 0.02% SA, 300 Gy γ rays + 0.03% SA treatments in the var. Gomati VU-89 and 100 Gy γ rays and 100 Gy γ rays + 0.01% SA treatments in the var. Pusa-578 induced a statistically significant increase in the mean number of pods per plant (**Tables 1**, **2**). The maximum increase in the mean number of pods per plant was noted with 100 Gy γ rays + 0.01% SA treatments in Gomati VU-89 (62.23) and Pusa-578 (42.63), respectively. The results revealed that only lower mutagen doses induced a statistically insignificant and significant decrease in the mean number of branches per plant in varieties Gomati VU-89 and Pusa-578, respectively (**Tables 1**, **2**). The minimum decrease in the mean number of branches per plant was recorded with 400 Gy γ rays + 0.04% SA and 200 Gy γ rays treatments in Gomati VU-89 (8.20) and Pusa-578 (10.40), respectively. The results revealed that all the mutagen doses induced a statistically insignificant increase in seed per pod with the highest increase in 0.01% SA treatment in the var. Gomati VU-89 (12.70) (**Table 1**). Except for 100 Gy γ rays, 0.01% SA, 100 Gy γ rays + 0.01% SA, all the mutagen doses induced a statistically insignificant increase in the mean number of SPP with the highest increase in 0.01% SA treatment in the var. Pusa-578 (10.90) (**Table 2**). The results revealed all the mutagen treatments except 200 Gy γ rays + 0.02% SA in var. Gomati VU-89 and 200 Gy γ rays + 0.02% SA and 300 Gy γ rays+0.03% SA in the var. Pusa-578 significantly decreased the mean SW in both varieties (**Tables 1**, **2**). The highest mean SW was noted in 200 Gy γ rays + 0.02% SA and 300 Gy γ rays + 0.03% SA treatments in the varieties Gomati VU-89 (14.80 g) and Pusa-578 (22.30 g), respectively. The highest increase in mean pod length was noted with 200 Gy γ rays + 0.02% SA and 200 Gy γ rays treatments in Gomati VU-89 (31.47 cm) and Pusa-578 (26.43 cm), respectively. The results revealed 100 Gy γ rays, 200 Gy γ rays, 300 Gy γ rays, 0.01% SA, 0.02% SA, 0.03% SA, 100 Gy γ rays + 0.01% SA, 200 Gy γ rays + 0.02% SA treatments in the var. Gomati VU-89 and 200 Gy γ rays, 0.01% SA, 0.02% SA, 100 Gy γ rays + 0.01% SA, 200 Gy γ rays + 0.02% treatments in the var. Pusa-578 induced a statistically significant increase in the mean pod length (**Tables 1**, **2**). Except for 200 Gy γ rays + 0.02% SA in the var. Gomati VU-89, and 400 Gy γ rays + 0.04% SA treatments in the var. Pusa-578, all the mutagen doses induced a statistically significant increase in the mean plant yield with a maximum increase in 100 Gy γ rays and 0.01% SA treatment in the varieties Gomati VU-89 (113.02 g) and Pusa-578 (100.54 g), respectively (**Tables 1**, **2**). All the lower and intermediate mutagen doses induced a statistically significant increase in the mean harvest index in both varieties, with the maximum increase in 100 Gy γ rays and 200 Gy γ rays treatment in the varieties Gomati VU-89 (40.53%) and Pusa-578 (37.42%), respectively (**Tables 1**, **2**).

**Table 1:** Estimates of mean and standard error (x̅ **±** SE) (n = 30) for quantitative traits in M_1_ generation of cowpea var. Gomati VU-89.

Treatments	PH	DF	DM	BPP	PL	PPP	SPP	SW	PY	HI
C	182.25^a^ ± 0.47	79.66^ab^ ± 0.30	150.93^a^ ± 0.29	10.73^a^ ± 0.10	28.72^f^ ± 0.15	59.20^ef^ ± 0.20	11.90^a^ ± 0.13	15.20^a^ ± 0.42	93.69^i^ ± 0.40	27.35^j^ ± 0.13
G1	180.14^abc^ ± 0.96	78.96^abc^ ± 2.49	149.46^a^ ± 0.61	9.46^ab^ ± 0.16	29.85^de^ ± 0.26	60.26^cde^ ± 0.44	12.40^a^ ± 0.20	14.10^cd^ ± 0.35	113.02^a^ ± 0.63	40.53^a^ ± 0.37
G2	178.58^bcde^ ± 1.19	79.96^ab^ ± 0.49	150.03^a^ ± 0.70	9.83^ab^ ± 0.18	30.51^bcd^ ± 0.28	62.20^a^ ± 0.52	12.30^a^ ± 0.24	14.20^bcd^ ± 0.3	107.87^cd^ ± 0.76	39.26^bc^ ± 0.35
G3	177.43^cdef^ ± 1.24	79.83^ab^ ± 0.50	149.66^a^ ± 0.98	9.03^bcd^ ± 0.18	30.27^cd^ ± 0.25	59.30^ef^ ± 0.49	12.26^a^ ± 0.24	14.30^bcd^ ± 0.34	103.87^f^ ± 0.58	38.10^d^ ± 0.35
G4	175.67^def^ ± 1.19	78.40^bc^ ± 0.51	148.93^a^ ± 1.03	8.53^cdef^ ± 0.17	29.25^ef^ ± 0.17	57.90^g^ ± 0.51	12.00^a^ ± 0.27	14.10^cd^ ± 0.30	97.96^g^ ± 0.49	35.58^fg^ ± 0.29
S1	181.95^ab^ ± 1.20	78.93^abc^ ± 0.47	149.93^a^ ± 0.71	9.23^bcd^ ± 0.18	30.61^bc^ ± 0.27	60.50^bcde^ ± 0.35	12.70^a^ ± 0.29	14.02^cd^ ± 0.28	111.02^b^ ± 0.79	39.73^ab^ ± 0.44
S2	180.30^abc^ ± 1.22	79.03^abc^ ± 0.50	151.46^a^ ± 0.81	8.90^cde^ ± 0.18	31.20^ab^ ± 0.26	60.70^bcd^ ± 0.42	12.30^a^ ± 0.26	14.50^bcd^ ± 0.25	109.00^c^ ± 0.65	38.73^cd^ ± 0.38
S3	178.83^abcd^ ± 1.22	79.46^abc^ ± 0.56	149.50^a^ ± 0.93	8.83^cde^ ± 0.19	29.88^de^ ± 0.21	59.70^de^ ± 0.43	12.20^a^ ± 0.23	14.62^bcd^ ± 0.26	106.33^de^ ± 0.66	36.22^ef^ ± 0.25
S4	176.60d^ef^ ± 1.24	78.80^abc^ ± 0.57	148.53a ± 0.98	8.60^cdef^ ± 0.19	28.88^f^ ± 0.16	60.10^cde^ ± 0.60	12.00^a^ ± 0.26	13.71^cd^ ± 0.37	98.80^g^ ± 0.42	35.79^ef^ ± 0.24
G1+S1	180.52^abc^ ± 1.11	80.23^a^ ± 0.54	150.83^a^ ± 1.02	9.33^ab^ ± 0.16	30.74^bc^ ± 0.28	62.23^a^ ± 0.32	12.10^a^ ± 0.27	14.03^cd^ ± 0.28	105.41^ef^ ± 0.65	36.59^e^ ± 0.38
G2+S2	177.30^cdef^ ± 1.11	79.36^abc^ ± 0.53	149.30^a^ ± 1.02	9.13^bcd^ ± 0.17	31.47^a^ ± 0.27	61.43^abc^ ± 0.34	12.00^a^ ± 0.30	14.80^a^ ± 0.27	107.62^cd^ ± 0.71	34.78^g^ ± 0.32
G3+S3	175.22^ef^ ± 0.87	79.16^abc^ ± 0.59	149.00^a^ ± 1.13	8.40^fgh^ ± 0.20	29.27^ef^ ± 0.16	61.70^ab^ ± 0.44	11.96^a^ ± 0.29	13.45^e^ ± 0.27	95.85^h^ ± 0.57	31.54^h^ ± 0.28
G4+S4	174.13^f^ ± 0.77	77.93^c^ ± 0.59	148.83^a^ ± 1.18	8.20^gh^ ± 0.18	28.96^f^ ± 0.15	58.20^fg^ ± 0.48	11.91^a^ ± 0.26	13.60^e^ ± 0.24	89.34^j^ ± 0.53	29.91^i^ ± 0.19

PH, plant height; DF, days to flowering; DM, days to maturity; PPP, pods per plant; BPP, branches per plant; SPP, seeds per pod; SW, seed weight; PL, pod length; PY, plant yield; HI, harvest index. #Means having same letter is not different at 5% level of significance, based on the DMRT. C, Control; G1, 100 Gy γ rays; G2, 200 Gy γ rays; G3, 300 Gy γ rays; G4, 400 Gy γ rays; S1, 0.01% SA; S2, 0.02% SA; S3, 0.03% SA; S4, 0.04% SA; G1 +S1, 100 Gy γ rays+0.01% SA; G2+S2, 200 Gy γ rays+0.02% SA; G3+S3, 300 Gy γ rays+0.03% SA; G4+S4, 400 Gy γ rays+0.04% SA.

**Table 2:** Estimates of mean and standard error (x̅ **±** SE) (n = 30) for quantitative traits in M_1_ generation of cowpea var. Pusa-578.

Treatments	PH	DF	DM	BPP	PL	PPP	SPP	SW	PY	HI
C	180.46^a^ ± 0.53	88.00^abcd^ ± 0.21	156.00^a^ ± 0.28	11.20^a^ ± 0.16	23.53^d^ ± 0.14	40.36^bc^ ± 0.18	9.46^de^ ± 0.14	22.97^a^ ± 0.14	79.90^f^ ± 0.32	28.79^f^ ± 0.14
G1	178.90^ab^ ± 1.00	88.53^abc^ ± 0.67	155.53^a^ ± 1.02	10.90^b^ ± 0.25	23.72^d^ ± 0.27	42.53^a^ ± 0.63	10.56^ab^ ± 0.22	21.70^b^ ± 0.24	97.81^b^ ± 1.01	34.28^c^ ± 0.36
G2	178.25^abc^ ± 1.12	89.43^a^ ± 0.69	155.43^a^ ± 1.12	10.40^c^ ± 0.31	26.43^a^ ± 0.37	41.73^ab^ ± 0.56	10.10^bcd^ ± 0.23	21.80^bc^ ± 0.35	91.45^c^ ± 0.84	37.42^a^ ± 0.41
G3	177.54^abc^ ± 0.91	88.43^abc^ ± 0.57	156.46^a^ ± 0.88	10.90^b^ ± 0.20	23.56^d^ ± 0.26	41.2^abc^ ± 0.44	9.80^cde^ ± 0.19	21.10^bcd^ ± 0.26	86.07^d^ ± 0.72	33.12^d^ ± 0.31
G4	176.10^bcd^ ± 0.83	87.8^abcd^ ± 0.48	155.00^a^ ± 0.79	10.80^b^ ± 0.18	23.12^d^ ± 0.23	40.53^bc^ ± 0.64	9.90^cde^ ± 0.19	20.96^cd^ ± 0.18	83.21^e^ ± 0.61	31.72^e^ ± 0.21
S1	180.26^a^ ± 1.14	87.53^abcd^ ± 0.70	156.06^a^ ± 1.13	10.70^b^ ± 0.34	25.82^ab^ ± 0.32	41.93^ab^ ± 0.38	10.90^a^ ± 0.23	21.04^c^ ± 0.25	100.54^a^ ± 1.02	36.42^b^ ± 0.35
S2	179.24^ab^ ± 1.18	86.43^d^ ± 0.64	156.80^a^ ± 1.16	11.1^a^ ± 0.37	24.70^c^ ± 0.29	41.26^abc^ ± 0.49	10.00^bcde^ ± 0.21	21.90^b^ ± 0.29	90.35^c^ ± 1.07	29.39^g^ ± 0.25
S3	178.11^abc^ ± 0.82	88.06^abcd^ ± 0.51	155.03^a^ ± 0.91	11.03^a^ ± 0.22	23.60^d^ ± 0.23	41.80^ab^ ± 0.58	9.70^cde^ ± 0.18	21.44^bcd^ ± 0.24	86.97^d^ ± 0.69	33.52^cd^ ± 0.29
S4	177.76^abc^ ± 1.22	89.00^ab^ ± 0.50	155.90^a^ ± 0.72	11.00^a^ ± 0.22	23.22^d^ ± 0.22	38.23^d^ ± 0.60	9.90^cde^ ± 0.18	20.89^d^ ± 0.21	74.70^g^ ± 0.54	32.85^d^ ± 0.28
G1+S1	179.44^ab^ ± 1.04	87.40^bcd^ ± 0.71	156.03^a^ ± 1.22	10.80^b^ ± 0.31	25.55^b^ ± 0.37	42.63^a^ ± 0.43	10.20^bc^ ± 0.24	22.10^b^ ± 0.30	96.09^b^ ± 0.96	34.38^c^ ± 0.44
G2+S2	177.36^abc^ ± 1.09	87.63^abcd^ ± 0.75	156.30^a^ ± 1.25	11.10^a^ ± 0.30	25.24^bc^ ± 0.35	41.00^abc^ ± 0.54	9.56^cde^ ± 0.20	22.20^ab^ ± 0.25	85.74^d^ ± 0.84	33.48^cd^ ± 0.41
G3+S3	175.49^cd^ ± 1.11	86.43^d^ ± 0.47	155.00^a^ ± 0.90	11.03^a^ ± 0.20	23.62^d^ ± 0.23	39.96^c^ ± 0.68	9.46^de^ ± 0.17	22.30^a^ ± 0.21	82.32^e^ ± 0.75	28.83^g^ ± 0.28
G4+S4	173.05^d^ ± 1.22	86.93^cd^ ± 0.47	154.80^a^ ± 0.86	11.0^a^ ± 0.19	23.29^d^ ± 0.21	37.83^d^ ± 0.48	9.36^e^ ± 0.15	22.0^bc^ ± 0.20	76.10^g^ ± 0.66	27.63^g^ ± 0.24

PH, plant height; DF, days to flowering; DM, days to maturity; PPP, pods per plant; BPP, branches per plant; SPP, seeds per pod; SW, seed weight; PL, pod length; PY, pant yield; HI, harvest index. #Means followed by the same letter is not different at 5% level of significance, based on the DMRT. C, Control; G1, 100 Gy γ rays; G2, 200 Gy γ rays; G3, 300 Gy γ rays; G4, 400 Gy γ rays; S1, 0.01% SA; S2, 0.02% SA; S3, 0.03% SA; S4, 0.04% SA; G1 +S1, 100 Gy γ rays+0.01% SA; G2+S2, 200 Gy γ rays+0.02% SA; G3+S3, 300 Gy γ rays+0.03% SA; G4+S4, 400 Gy γ rays+0.04% SA.

### Correlation analysis

**Figure 5:**
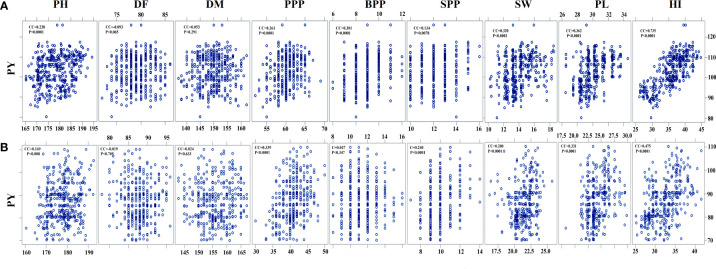
Estimates of Pearson’s correlation coefficients between plant yield (PY) and yield- attributing traits **(A)** var. Gomati VU-89 and **(B)** var. Pusa-578 (n = 30 for each character). PH, plant height; DF, days to flowering; DM, days to maturity; PPP, pods per plant; BPP, branches per plant; SPP, seeds per pod; SW, seed weight; PL, pod length; PY, plant yield; HI, harvest index (n = 30 for each character).

Pearson’s correlation analysis revealed that PY is positively correlated with SW, SPP, PPP, and BPP (*P* < 0.001) and negatively correlated with DF (*P* < 0.705). In the var. Gomati VU-89, PY revealed a significant positive correlation with the HI (0.735), PPP (0.261), PL (0.362), PH (0.238), BPP (0.301), and SW (0.320) and an insignificant correlation with SPP (0.134), DF (-0.093), and DM (0.053). In the var. Pusa-578, PY showed a significant positive correlation with PPP (0.339), PL (0.331), SPP (0.240), SW (0.200), PL (0.331), and HI (0.475) and an insignificant correlation with PH (0.169), BPP (0.047), DF (-0.019), and DM (0.024) (**Supplementary Table 1**; **Figure 5**). Correlation analysis revealed that PL and PPP are the most important yield-attributing traits in Gomati VU-89 and Pusa-578, respectively. The contribution of these nine yield components in decreasing order was HI > PL > SW > BPP > PPP > PH > SPP > DF > DM in the var. Gomati VU-89 and HI > PPP > PL > SPP > SW > PH > PH > BPP > DM > DF in the var. Pusa-578.

### Path analysis

**Figure 6:**
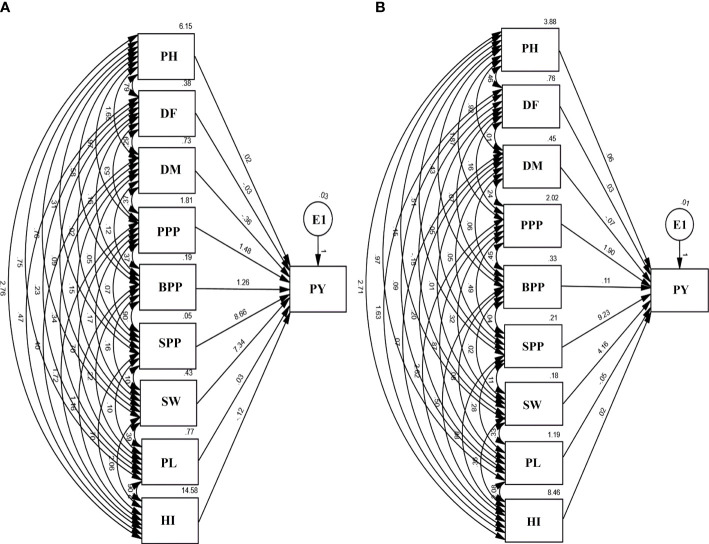
Path diagram **(A)** var. Gomati VU-89 and **(B)** var. Pusa-578 showing the interrelationship between PY and yield attributes viz., PH, plant height; DF, days to flowering; DM, days to maturity; PPP, pods per plant; BPP, branches per plant; SPP, seeds per pod; SW, seed weight; PL, pod length; PY, plant yield; HI, harvest index (n = 30 for each character) and E1: residual factors.

Path analysis revealed that SPP and SW had a maximum positive direct effect on the PY. However, PL and DM revealed a negative direct effect on yield. E1 in the diagram represents residual factors, unaccounted for and independent of the other variables. The contributions of trait toward yield showed an increasing trend, i.e., DM< DF< PH< PL< PL< HI< BPP< SW< SPP in the var. Gomati VU-89 and BPP< DM< PL< HI< DF< PH< PPP< SW< SPP in the var. Pusa-578 (**Figure 6**).

#### Plant yield vs. plant height

The direct effects of PH on PY was 0.02 and 0.6 in Gomati VU-89 and Pusa-578, respectively. It had positive indirect impact on PY through DF (0.79 and 0.46), DM (1.65 and 0.92), PPP (0.97 and 1.87), BPP (0.58 and 0.43), SPP (0.31 and 0.51), SW (0.76 and 0.15), PL (0.75 and 0.97), and HI (2.76 and 2.71) in the var. Gomati VU-89 and var. Pusa-578, respectively (**Figure 6**).

#### Plant yield vs. days to flowering

The direct effect of DF on PY was -0.03 and 0.03 in the var. Gomati VU-89 and var. Pusa-578, respectively. It had a positive indirect impact on PY through DM (0.29 and 0.01), PPP (0.53 and 0.16), BPP (0.16 and 0.7), SPP (0.02 and 0.05), SW (0.09 and 0.15), PL (0.23 and 0.09), and HI (0.47 and 1.63) in the var. Gomati VU-89 and var. Pusa-578, respectively (**Figure 6**).

#### Plant yield vs. days to maturity

The direct effect of DM on PY was -0.36 and -0.07 in the var. Gomati VU-89 and var. Pusa-578, respectively. It had an indirect impact on PY through PPP (0.37 and 0.24), BPP (0.12 and 0.06), SPP (0.05 and 0.05), SW (0.15 and 0.01), PL (0.34 and 0.20), and HI (0.40 and 0.07) in the var. Gomati VU-89 and var. Pusa-578, respectively (**Figure 6**).

#### Plant yield vs. pods per plant

The direct effect of PPP on PY was 1.48 and 1.90 in the var. Gomati VU-89 and var. Pusa-578, respectively. It had a positive indirect impact on PY through BPP (0.37 and 0.45), SPP (0. 07 and 0.49), SW (0.17 and 0.32), PL (0.70 and 0.87), and HI (1.72 and 2.62) in the varieties Gomati VU-89 and Pusa-578, respectively (**Figure 6**).

#### Plant yield vs. branches per plant

The direct impact of BPP on PY was 1.26 and -0.11 in the var. Gomati VU-89 and var. Pusa-578, respectively. BPP also positively impacted PY through PL (0.22 and 0.08) in Gomati VU-89 and Pusa-578, respectively. A positive indirect impact on PY was shown by BPP through HI (1.16 and 0.50), SPP (0.06 and 0.04), and SW (0.16 and 0.02) in the var. Gomati VU-89 and var. Pusa-578, respectively (**Figure 6**).

#### Plant yield vs. seeds per pod

In the var. Gomati VU-89 and Pusa-578, the direct impact of SPP on PY was 8.66 and 9.23, respectively. It also had a positive indirect impact on PY through SW (0.10 and 0.11), PL (0.10 and 0.28), and HI (0.70 and 0.88) in the var. Gomati VU-89 and var. Pusa-578, respectively (**Figure 6**).

#### Plant yield vs. seed weight

The direct impact of SW on PY was 7.34 and 4.16 in the var. Gomati VU-89 and var. Pusa-578, respectively. SW also showed a considerable positive indirect impact on PY through PL (0.39 and 0.33) and a strong positive indirect impact on PY through HI (2.06 and 0.36) in the var. Gomati VU-89 and var. Pusa-578, respectively (**Figure 6**).

#### Plant yield vs. pod length

The direct impact of PL on PY was 0.03 and -0.05 in the varieties Gomati VU-89 and Pusa-578. PL also showed a strong positive indirect impact on PY through HI (2.06 and 2.08) in the var. Gomati VU-89 and var. Pusa-578, respectively (**Figure 6**).

#### Plant yield vs. harvest index

The direct impact of the HI on PY was -0.12 and 0.02 in the var. Gomati VU-89 and var. Pusa-578, respectively (**Figure 6**).

### Multivariate analysis

#### Hierarchical cluster analysis

**Figure 7:**
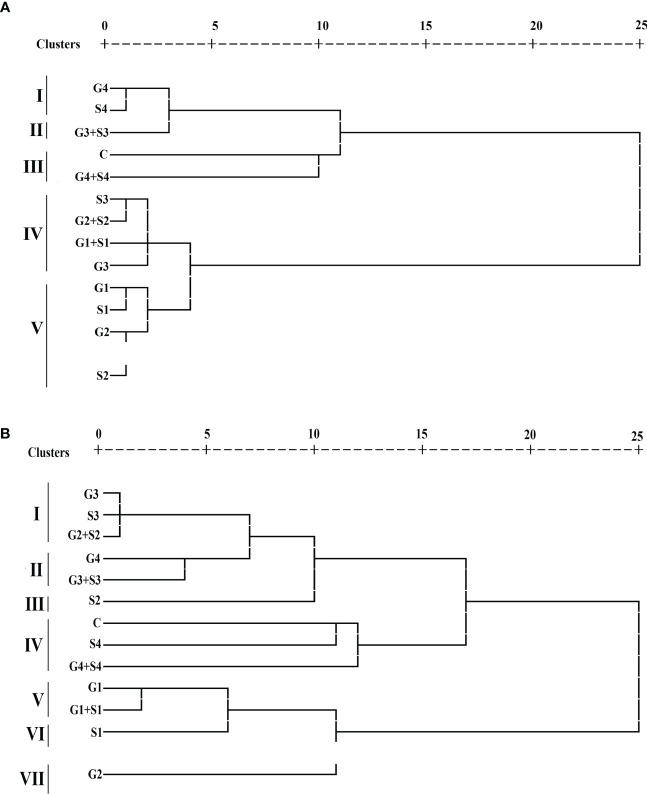
Dendrogram analysis **(A)** var. Gomati VU-89 and **(B)** var. Pusa-578 of control and mutagen-treated population based on 10 quantitative traits (n = 30). C, Control; G1, 100 Gy γ rays; G2, 200 Gy γ rays; G3, 300 Gy γ rays; G4, 400 Gy γ rays; S1, 0.01% SA; S2, 0.02% SA; S3, 0.03% SA; S4, 0.04% SA; G1 +S1, 100 Gy γ rays+0.01% SA; G2+S2, 200 Gy γ rays+0.02% SA; G3+S3, 300 Gy γ rays+0.03% SA; G4+S4, 400 Gy γ rays+0.04% SA.

HCA categorized treated and control plants into separate clusters, each with cluster size ranging from 1 to 4 in both varieties. Each 12 treated and 1 untreated population were grouped into five and seven clusters in the varieties Gomati VU-89 Pusa-578, respectively (**Figure 7**). In the var. Gomati VU-89, group I included 400 Gy γ rays and 0.04% SA populations, group II comprised 300 Gy γ rays + 0.03% SA population, group III comprised control and 400 Gy γ rays + 0.04% SA populations, group IV comprised 0.03% SA, 200 Gy γ rays + 0.02% SA, 100 Gy γ rays + 0.01% SA, and 300 Gy γ rays populations, and group V constituted 100 Gy γ rays, 0.01% SA, 200 Gy γ rays, and 0.02% SA populations. In the var. Pusa-578, group I consisted of 300 Gy γ rays, 0.03% SA and 200 Gy γ rays + 0.02% SA populations; group II comprised 400 Gy γ rays and 300 Gy γ rays + 0.03% SA populations; group III consisted of 0.03% SA population; group IV included control, 0.04% SA, and 400 Gy γ rays + 0.04% SA populations; group V composed of 100 Gy γ rays and 100 Gy γ rays + 0.01% SA population; group VI consisted of 0.01% SA population; and group VII consisted of 200 Gy γ rays population. Among mutagenized plants, 0.02% SA and 200 Gy γ rays populations were most diverged from untreated populations in Gomati VU-89 and Pusa-578, respectively.

Characteristic means of quantitative phenotypic traits of both treated and untreated populations were shown by three clusters I, II, and III (**Supplementary Table 2**). Among the clusters, cluster II showed maximum mean values for PPP (60.27 and 41.93), SPP (12.40 and 10.90), SW (15.10 and 22.04), PL (29.85 and 25.82), PY (113.02 and 100.54), and HI (40.54 and 36.42%) in the varieties Gomati VU-89 and Pusa-578, respectively. Therefore, the population that lies in cluster II could be used for isolating high-yielding mutant lines. The highest intercluster distance was noted between clusters I and II in the varieties Gomati VU-89 (19.28) and Pusa-578 (21.21), respectively (**Supplementary Table 3**).

The interpopulation dissimilarity matrix revealed the highest Euclidean distance between 400 Gy γ rays + 0.04% SA and 100 Gy γ rays populations in the var. Gomati VU-89 (26.88) and 400 Gy γ rays + 0.04% SA and 0.01% SA populations in the var. Pusa-578 (27.47) indicate a dissimilar population. The minimum Euclidean distance was noted between 0.01% SA and 100 Gy γ rays populations in the var. Gomati VU-89 (3.03) and 0.03% SA and 300 Gy γ rays population in the var. Pusa-578 (2.09) indicates a similar population. The most distanced population from control was 100 Gy γ rays and 0.01% SA population in the varieties Gomati VU-89 (23.68) and Pusa-578 (22.27), respectively (**Supplementary Table 4**).

#### Principal component analysis

**Figure 8:**
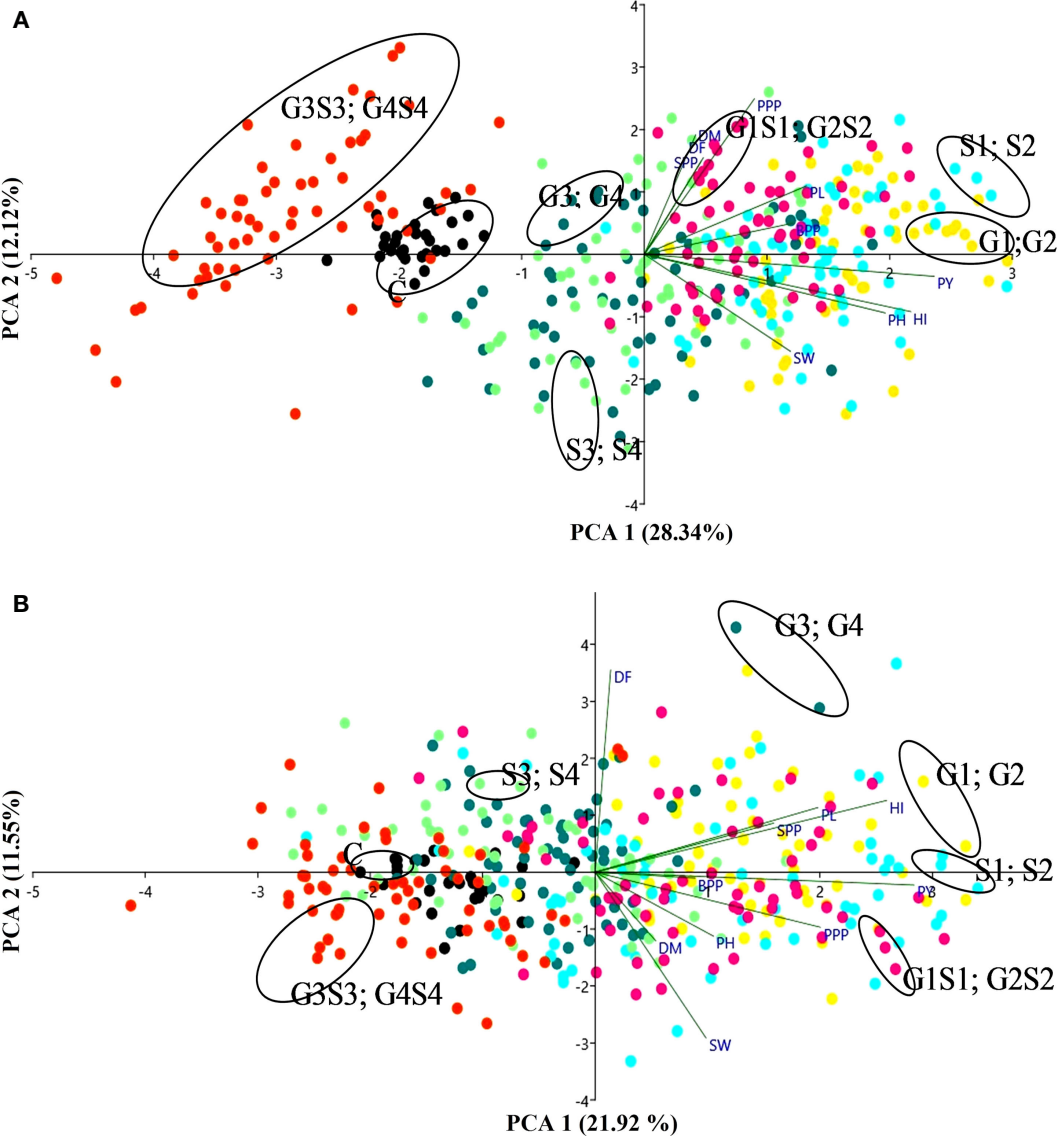
The biplots of principal component analysis **(A)** var. Gomati VU-89 and **(B)** var. Pusa-578 compare the effects of mutagens on mean yield and yield attributes (n = 30). PH, plant height; DF, days to flowering; DM, days to maturity; PPP, pods per plant; BPP, branches per plant; SPP, seeds per pod; SW, seed weight; PL, pod length; PY, plant yield; HI, harvest index. C, Control; G1, 100 Gy γ rays; G2, 200 Gy γ rays; G3, 300 Gy γ rays; G4, 400 Gy γ rays; S1, 0.01% SA; S2, 0.02% SA; S3, 0.03% SA; S4, 0.04% SA; G1 S1, 100 Gy γ rays+0.01% SA; G2S2, 200 Gy γ rays+0.02% SA; G3S3, 300 Gy γ rays+0.03% SA; G4S4, 400 Gy γ rays+0.04% SA.

The PCA confirmed that lower and intermediate mutagen–treated populations viz., 100 Gy γ rays, 200 Gy γ rays; 0.01% SA, 0.02% SA; 100 Gy γ rays + 0.01% SA, 200 Gy γ rays + 0.02% SA and higher mutagen–treated population viz., 300 Gy γ rays, 400 Gy γ rays; 0.03% SA, 0.04% SA; 300 Gy γ rays + 0.03% SA formed their separate clusters as represented by different colored dots in a biplot (**Figure 8**). The PCA showed a total 40.47% (PC1 = 28.34%; PC2 = 12.12%) and 33.47% (PC1 = 21.92%; PC2 = 11.55%) variability of the data in the var. Gomati VU-89 and var. Pusa-578, respectively. PY (0.525 and 0.532) contributed more to the variation followed by HI (0.483 and 0.486), PH (0.436 and 0.197), PL (0.293 and 0.371), and BPP (0.269 and 0.166). SW (0.265 and 0.184) and PPP (0.199 and 0.375) had the highest loadings in PC1 in the varieties Gomati VU-89 and Pusa-578, respectively. Characters that contributed to the second component included PPP (0.554 and -0.181), DM (0.425 and -0.226), DF (0.344 and 0.666), SPP (0.331 and 0.161), and PL (0.243 and 0.212) in the varieties Gomati VU-89 and Pusa-578, respectively. PY and SW showed maximum positive and minimum negative loading on PC1 and PC2 in the var. Gomati VU-89 (0.525 and -0.345) and var. Pusa-578 (0.532 and -0.545), respectively (**Supplementary Table 5**). For PC2, DM revealed positive loading in var. Gomati VU-89 (0.425) and negative loading in the var. Pusa-578 (-0.226). Substantial positive correlations between PY and PPP, SPP, and BPP were recorded in both varieties. Based on the distribution pattern of mutagen-treated populations in the biplot, it can be concluded that mutagen doses induced substantial variation in the quantitative traits (**Figure 8**). PY was the most important trait contributing for the overall variability observed among the putative mutants. In the present study, PCA revealed that the first two principal components contributed maximum number of traits toward variability.

## Discussion

### Nitrate reductase activity, chlorophyll, and carotenoid contents assessment in control and treated plants

Nitrate reductase, a molybdoenzyme with a molybdenum atom linked to two sulfur atoms of molybdopterin, is involved in producing nitric oxide in plants. Nitric oxide is vital in germination, growth, apoptosis, stomatal regulation, and stress tolerance (Wendehenne, 2011; Sanz-Luque et al., 2015). In the present study, a linear decrease in Nitrate Reductase (NR) activity in the mutagen- treated seedlings was in propinquity with the results obtained by Laskar et al. (2015). However, the mechanism behind this inhibition is not fully understood, and several workers attributed mutagen-induced inhibition and metabolic dysfunctions of specific enzyme proteins to the decreased NR activity (Hopkins, 1995).

Chlorophyll plays a vital role in photosynthesis and influences key processes of plant growth and development (Li et al., 2018). Chlorophyll synthesis is a complex process involving several enzymes and steps like insertion of Mg^2+^ into protoporphyrin IX that affect chlorophyll biosynthesis (Tanaka and Tanaka, 2007). Any obstructive enzymatic reaction or step may lead to reduced chlorophyll content (Willows and Hansson, 2003). In the present study, a decrease in mean chlorophyll contents in mutagenized populations was in agreement with the findings of Borzouei et al. (2010) in *Triticum aestivum* and Verma et al. (2010) in *Euryale ferox*. The diminution in chlorophyll content may be due to mutagen-induced loss of function in genes related to chlorophyll synthesis. Moreover, reduction in chlorophyll content may be due to the gamma radiation–induced dephytolization and pheophytinization that eventually results in inhibition of chlorophyll synthesis (Saha et al., 2010). Mutagens, especially γ radiations, interact with the atoms and molecules and generate highly reactive free radicals that destroy or modify photosynthetic pigments, thylakoid membranes, the antioxidative system, and essential constituents of a cell (Kovacs and Keresztes, 2002; Kulandaivelu and Noorudeen, 1983; Wi et al., 2007). Kim et al. (2004) reported that γ rays induced prominent structural changes in chloroplasts, such as inhibition of senescence and dedifferentiation into the agranal stage. Kiong et al. (2008) reported that the reduction in chlorophyll b is mainly due to selective degradation of chlorophyll b.

Carotenoids act as accessory pigments with vital roles in the light-harvesting process, quenching excess light energy, scavenging free radicals, and creating a plant defense system (Fukuzawa et al., 1998; Ramel et al., 2012; Hashimoto et al., 2016). The results revealed a substantial decrease in carotenoid contents in the treated population of both varieties. The results followed the findings of Amin et al. (2019) in mutagen-treated black cumin populations. The reduction in carotenoid contents may be attributed to mutagen-induced loss of function of carotenoid-synthesizing genes. The reduced carotenoids in treated seeds could reduce the light-harvesting and photosynthetic capacity. In general, alterations in the enzymes involved in the carotenoid synthesis could be considered one of the physiological effects caused by gamma rays and SA, which leads to decreased pigment contents in mutagenized populations.

### Quantitative traits and their role in total plant yield

Small additive effects of multiple genes govern quantitative traits, and their expression is also influenced by environmental factors. Hence, statistical analysis of quantitative traits is required to visualize the data significance. In the present study, enhanced quantitative traits of lower and intermediate mutagen–treated plants could be attributed to the induction of desirable mutations. However, decreased quantitative traits in plants raised from seeds treated with higher combined mutagen doses might be ascribed to the synergistic effect of the combined mutagens. It is recommended to expose the plant material to individual mutagens to evade the deleterious effects of combined mutagens. Additionally, it will facilitate a chance for the second mutagen to target the DNA region left unaltered by the first mutagen, resulting in a high frequency of mutations.

In the present study, mutagen-induced reduction in plant height may be ascribed to a diminution of mitotic activity of meristematic tissues, cell length, cell number, and phytohormones (Austin et al., 1980; Cheng et al., 2019). The results were in propinquity with the findings obtained in lentil (Laskar et al., 2018), rapeseed and mustard (Zeng et al., 2011; Wei et al., 2018; Channaoui et al., 2019), and coriander (Kumar and Pandey, 2019). Mutagen-induced reduction in plant height is a crucial trait from the perspective of plant breeders. This is because short-statured plants have improved architecture, yield, harvest index, and lodging resistance and are less influenced by the fast-moving winds that are quite prevalent in the flowering season of cowpea. More and Malode (2016) also reported increased yield in dwarf mutants of canola treated with Ethyl methane sulphonate (EMS). Further, one could recall that the use of dwarfing genes was a crucial factor in accomplishing the green revolution (Khush, 2001). In the present study, mutagen doses positively affected flowering time and could lead to the isolation of early-flowering mutants in subsequent generations. The results followed the findings of Gnanamurthy and Dhanavel (2014), which reported a reduction in flowering time in the mutagenized cowpea population. Flowering time, the main modulator of plant growth, is influenced by environmental factors such as temperature, photoperiod, nutrient availability, light quality, and intensity (Pouteau et al., 2004). Therefore, it is difficult to assess the cause of reduction in flowering time in the plants raised from mutagen-treated seeds. Early flowering offers adequate time for pod filling, resulting in improved seed yield. Earliness is most commonly altered in mutagenesis programs, and many early maturing mutants have been developed (Malek and Monshi, 2009). In the present study, few early maturity mutants have been isolated in the treated population. These putative mutants mature earlier than the wild-type lines and can escape detrimental heat stress at the pod-filling stage usually encountered in cowpea-growing areas. Moreover, early-maturing putative mutants are also good at escaping insect damage and averting rise in the insect population due to the shorter reproductive phase duration (Jackai, 1982). Such early maturing putative mutants are also suitable for plantations in areas receiving short rainfall. The diversity in terms of genetic and physiological properties of the early-flowering and maturing putative mutants arising from common ancestry accentuates the importance of induced mutagenesis in broadening the genetic base of cowpea. Reduction in the maturity period has also been reported earlier in γ-irradiated cowpea (Adekola and Oluleye, 2007; Horn et al., 2016). These mutants were found to have a mutational variation in vernalization and/or photoperiodic response. Pods per plant are one of the most crucial yield attributes that meticulously correlate with plant yield. The number of pods per plant is considered one of the most reliable bases for predicting yield in grain legumes; an increase in pods per plant in lower and intermediate dose–treated populations may lead to the isolation of high-yielding mutants in subsequent generations. The stimulatory effects of the lower and intermediate mutagen doses may be attributed to an augmented pod per plant. The results followed the findings of Essel et al. (2015) that reported an increased number of pods in lower doses and a reduced number of pods at higher doses of colchicine in cowpea. Omosun et al. (2021) also reported increased pods per plant in the pigeon pea accessions treated with 0.03% and 0.04% EMS. The increased pods is an vital character from the perspective of plant breeders, and putative mutants with augmented pods are a vital genetic resource for future breeding programs. In the present study, all mutagen doses negatively impacted the number of branches. The decreased number of branches per plant might be due to cellular divisions at a low rate, reduced photosynthetic activities, and synthesis of growth regulators. Putative mutants with decreased branches were also found to have few pods, flowers, fodder, and seed yield. Contrary to the present study, mutagen doses caused a substantial increase in the number of branches reported earlier in mutagenized coriander (Kumar and Pandey, 2019) and tomato (Akhtar, 2014). The number of seeds per pod plays an important role in developing high-yielding mutant lines, and working on the improvement of this trait could be an effective way to augment yield. Therefore, seeds per pod are a vital component of yield traits in grain legumes; mutants with more seeds per pod reflect higher yields than mutants with fewer seeds (Zhou et al., 2005). In the present study, we noted that lower treatments showed a substantial increase in the seeds per pod, indicating that γ rays and SA have a promoter effect on seed formation. This may be due to mutagen-altered gene expression that governs seed formation or increased activity of growth regulators that impact seed production (Omosun et al. (2021)). Mutagen-induced non-significant increase in seeds has been reported by Horn et al. (2016) in cowpea, Khursheed et al. (2018) in faba bean, and Laskar and Khan (2017) in lentil. In the rapidly increasing population era, there will be an ever-increasing pressure on food production, and more food will be needed to feed the burgeoning population. Hence, increasing food production is a major task for the 21st century. Since the world’s food calories is obtained mostly from seed, one way to accomplish this task is to develop mutant lines with more and larger seeds. Furthermore, seed weight in grain crops is considered as one of the most important agronomic traits in plant breeding programs. During the domestication of crop plants, seed weight has been given a primary importance in selection (Linkies et al., 2010; Li and Li, 2014; Ge et al., 2016; Li and Li, 2016). In the present study, mutagen doses of γ rays and SA decreased the mean seed weight. Generally, seed weight is negatively correlated with pods per plant and seeds per plant. Therefore, it is not surprising to record a reduction in seed weight in a mutagen-treated population. The decrease in seed weight may be attributed to the inhibitory effects of mutagen doses. The results were in propinquity with the findings of Amin et al. (2015) in lentil. In the present study, the increased pod length may be attributed to the desirable mutations in the treated plants. Longer pods can accommodate more seeds, leading to enhanced yield (Omosun et al. (2021)). Mutagen induced increases in the mean length of pods have been reported in cowpea genotypes treated with lower doses of γ rays (Horn et al., 2016).

Yield is an essential agronomic trait, and its enhancement is the ultimate aim of plant breeders and farmers (Omosun et al. (2021)). To boost the crop yield and farmers’ income, creating high- yielding cowpea varieties is necessary. In grain legumes, plant yield is determined by yield-attributing traits such as branches per plant, pods per plant, seeds per plant, and seed weight. The proportional increases and decreases in the plant yield, with increasing γ rays and SA doses reported in this study, were similar to the findings of Kumar et al. (2010) and Horn et al. (2016) in cowpea. Plant yield is a polygenic trait with a complex inheritance, making it difficult to determine its deviation from the control. Aside from random mutations, the influence of environmental flux on gene expression and the lack of whole genome sequencing data of cowpea are other obstacles in identifying the actual mutations that led to deviation from control plants. The ratio of seed yield to total dry weight defines the harvest index and signifies its capacity to allocate photosynthetic assimilates into grains (Donald and Hamblin, 1976; Sinclair, 1998; Wnuk et al., 2014). Plant yield is determined by two key components: biomass and harvest index. Biomass indicates the capability of producing an adequate amount of agricultural residue, while the harvest index indicates the capacity to allocate biomass to the desired harvestable product, such as grains in food legumes. Allocation of biomass or photosynthates toward desired harvestable product could play a vital role in obtaining a good yield and determines how plant biomass is converted to seed yield (Jack et al., 2014 (Chen et al., 2021). Allocation of photosynthates determines the overall seed yield and the commercial worth of grain crops. Hence, evaluation of the harvest index is imperative for harnessing the full genetic potential of legumes (Adeyanju, 2009). The harvest index also reveals the physiological efficiency in the conversion of dry matter into economic yield (Sharifi et al., 2009). An increase in the harvest index is associated with an increase in the economic portion of the crops (Sharma-Natu and Ghildiyal, 2005). In other words, a low harvest index may be attributed to low seed yield in food legumes. The harvest index plays a vital role in the augmentation of yield, which could help in accomplishing the feeding demands of the burgeoning population (Asefa, 2019). In the present study, the harvest index values indicated that only 30%–40% of the photosynthates were translocated to seeds in both varieties. The increased harvest index in the lower and intermediate treatments may be attributed to decreased branching, as secondary branches produce less seed than main stems. Laskar and Khan (2017) reported similar findings of an increased harvest index in lentil cultivars treated with lower doses of γ rays and hydrazine hydrates. Putative mutants showing a higher harvest index can be considered ideal genotypes for selection to improve yielding potential. Different putative mutants showed variation in the harvest index, which may be attributed to the physiological effects of γ rays and sodium azide. Increasing crop yield is required to supply sustained food and nutrition to the rapidly increasing world population. However, without increasing the harvest index, improvement in yield could not be accomplished. Therefore, the harvest index plays a critical role in enhancing crop productivity and may be considered selection criteria in crop improvement programs (Asefa, 2019).

### Correlation analysis: association of yield with its component traits

Correlation analysis plays a pivotal role in determining the association between different yield- attributing traits (Sandhu and Gupta, 1996; Sinha et al., 2001; Rameeh, 2016). The role of mutagens in altering correlations between traits has also been reported earlier in several cultivars (Toker and Cagirgan, 2004; Shin et al., 2011). The apparent differences in the correlation coefficient for different traits may be due to the pleiotropic effects of mutated genes. Yield enhancement, a prime goal of the crop improvement programs, requires a broader understanding of the relationship between yield and yield-contributing traits. The yield was treated as a resultant variable in the present study, and other traits were estimated as causal variables. There were 8 of the 10 component characters studied that exhibited significant positive associations with plant yield. The significant and positive correlation observed between plant yield and yield-contributing traits may be because these traits are vital determinants of plant yield. This showed that enhancement of these traits would result in better grain yield. Based on the strength of correlations, these six characters were ordered as the harvest index, followed by pod length, seed weigth, branches per plant, pods per plant, plant height, seeds per pod and days to maturity could be used as selection criteria in advanced generations for yield enhancement. However, plant yield correlated negatively with days to flowering in both varieties. Fewer days to flowering tend to have higher yields as early flowering helps the cowpeas evade detrimental heat stress during the reproductive phase. These findings also conformed to previous studies (Laskar and Khan, 2017) in which days to flowering were negatively associated with plant yield. Considerable positive phenotypic correlations for plant yield and pod length; plant yield and pods per per plant indicated that yield might be enhanced through the direct selection of these yield attributes.

Further direct selection for putative mutants bearing more pods and longer pods can provide better results for the improvement of yield in cowpea. Similar results have been reported by Aminu et al. (2016) while studying the correlation analysis in okra. Similarly, a strong positive correlation between plant yield and seed weight implies that plants with augmented seed weight would be more productive. Laskar and Khan (2017) also reported similar findings in lentils. The results revealed that the harvest index had a positive association with plant yield. Similar findings were reported by Marri et al. (2005), which also found a strong and positive correlation between yield and the harvest index. The present study revealed that there is scope for simultaneous improvement of these traits through selection. However, it is important to mention that correlation analysis produces fallacies about the trait association and is not enough to determine the direct and indirect impact of yield-attributing traits. Therefore, path analysis, a biometric technique, is important in assessing the exact picture of the relationship between different traits.

### Path analysis: direction and degree of trait associations

Path analysis determines the cause-and-effect relationship between the yield and its component traits. It enables the breeders to visualize the association between traits by evaluating correlation coefficients into direct and indirect effects. It also identifies the traits that significantly affect yield for potential use as selection criteria. Teodoro et al. (2014) reported that path analysis provides a broader understanding of the direction and degree of trait associations. Path analysis revealed that all the yield-contributing traits except pod length and branches per plant positively impacted the plant yield in varieties Gomati VU-89 and Pusa-578, respectively. In the present study, the trait of maximum influence on plant yield was the seeds per pod, as it depicted the highest direct effect on plant yield and simultaneously caused a high indirect effect on the pod number. This trait had an indirect effect *via* the harvest index (that, in turn, depends on the dry matter) on plant yield since its coefficient (0.70 and 0.88) was superior to the residual effect (0.3 and 0.01). This reflects that mutants with high dry matter per plant could divert more assimilates to seeds and increase yield. Since biomass is a product of leaves, branches, and plant height, these traits could, directly and indirectly, contribute to higher seed yield. Seeds per pod can be considered a trait of interest in indirect selection in cowpea breeding programs. The results followed Magashi et al. (2017) findings that reported crops with more seeds per pod would be expected to have higher yield.

Similarly, Silva et al. (2014) reported seeds per plant were the main component having a direct effect, while pods per plant showed an indirect effect on soybean yield. In addition to the seeds per pod, seed weight and pods per plant showed a positive direct impact on the yield, and hence, plants with more pods yield higher than plants with fewer pods. Elliott et al. (2008) also found that heavier seeds show high germination and shoot weight and higher yield than lighter seeds. Salehi et al. (2010) also reported positive and significant correlations between the number of seeds per pod, the number of pods per plant, and pod length, with grain yield in common bean. The combined contributions of either pods per plant and seed weight or seeds per pod and seed weight indicated that improving these characters could enhance plant yield. Days to flowering and days to maturity were positively and significantly correlated with plant yield; however, their direct impact was negative, depicting that indirect impact would be the cause of the correlation. In this state, the indirect causal factors should be considered simultaneously for selection. Hence, it is recommended to consider the traits that show a high indirect impact on plant yield. The residual effect (0.3 and 0.01) depicted that traits, which are included in the path analysis, explained 70% and 99% of the total variation on the dependent variable, i.e., plant yield in the varieties Gomati VU-89 and Pusa-578, respectively (the rest, 30% and 1%, was the contribution of other factors, such as traits not studied).

### Multivariate analysis: an effective tool in plant breeding programs

The multivariate analysis is useful for assessing the degree of divergence between mutagenized populations or putative mutants. Multivariate statistical techniques are also used to simultaneously analyze multiple measurements on each treated population. Irrespective of the data, whether morphological, biochemical, or molecular, multivariate statistical techniques are widely used in genetic diversity analysis. Among the multivariate techniques, PCA and HCA are useful in selection of mutants (Mohammadi and Prasanna, 2003). PCA and HCA are also considered the main tools for determining the relatedness and categorization of mutants based on quantitative data (Malek et al., 2014). In the present study, cluster analysis helped us in forming clusters for grouping putative mutants with similar features and vice versa. In any plant breeding experimentation, it is crucial to know plant traits that explain maximum variability. Therefore, PCA and HCA were conducted to run a classification analysis on the putative cowpea mutants using descriptive statistics and to understand the association of various characters. PCA and HCA, as described below, enabled us to categorize the putative mutants into distinct classes based on their genetic diversity.

#### Hierarchical cluster analysis: classifying putative mutants

In the present study, HCA was used to classify putative mutants into separate clusters based on genetic diversity among their quantitative traits. HCA categorized mutagenized populations into separate five clusters that significantly deviated from the respective controls in both varieties. HCA divided putative mutants into five and seven main clusters in Gomati VU-89 and Pusa-578 based on phenotypic traits. Clustering of putative mutants based on studied traits is presented in **Figure 7**. HCA grouped putative cowpea mutants into 12 clusters (5 clusters in the var. Gomati VU-89 and 7 clusters in the var. Pusa-578). In the var. Gomati VU-89, group I comprised of 400 Gy and 0.04% SA-treated populations and plants were short-statured, and low yielding as compared to all other genotypes of groups II, III, and V. Similarly, group II comprised of the 300 Gy + 0.03% SA population were having long pods, bold seeds, and higher yielding. Group III comprised of control, and the 400 Gy + 0.04% SA population were tall with a low pod set and total plant yield, while group IV comprised of 0.03% SA, 200 Gy + 0.02% SA, 100 Gy + 0.01% SA, and 300 Gy populations were with more number of pods and highest yield. Group V comprised 100 Gy, 0.01% SA, 200 Gy, and 0.02% SA populations were high yielding with desired traits. In the var. Pusa-578, a group I consisted of 300 Gy, 0.03% SA, and 200 Gy + 0.02% SA populations were tall, a low pod set, and low yielding, group II comprised of 400 Gy and 300 Gy + 0.03% SA populations were mostly plants with less number of seeds per pod that eventually resulted in low yield, group III consisted of the 0.03% SA population were low-yielding plants, group IV included control, 0.04% SA and 400 Gy + 0.04% SA populations were tall with fewer flowers that resulted into a low pod set and plant yield, group V composed of 100 Gy and the 100 Gy + 0.01% SA population were plants with more flowers per plant, more pod set, and high yield, group VI consisted of the 0.01% SA population were plants with bold-seeded pods, and group VII consisting of the 200 Gy population were plants with long pods. 0.02% SA and 200 Gy populations were most diverged from control populations in Gomati VU-89 and Pusa-578, respectively. The genetically similar and divergent mutagenized populations were distinctly separated and classified within the same and different clusters. The clustering of mutants based on their similarity/dissimilarity revealed that mutants grouped in different clusters are ideal for breeding programs aimed at broadening genetic variability. In induced mutagenesis, proper selection of mutant lines is achieved by selecting the most distinct cluster with respect to the control cluster. The intercluster distance determines the variability spectrum; the more distanced clusters indicate a broader spectrum of variability in the segregating generation and vice versa. In the present study, cluster II, the maximally contributing cluster, should be selected for further selection to broaden variability. The present study revealed that HCA allowed us to place the putative mutants and control plants in different clusters based on quantitative traits. Therefore, HCA can be considered an effective tool for sorting putative mutants and enables breeders to select the base material to design future breeding strategies in cowpea. In addition, it is imperative that while selecting base material, genetic barriers, and breeding methods must be given proper importance to achieve the desired goals of plant breeding.

#### Principal component analysis: assessing comparative contribution of traits toward variability 

In cowpea, yield is the cumulative effect of many yield-contributing traits. Different characteristics such as pods per plant, seeds per pod, pod length, and seed weight assume vital importance and must be assessed for a contribution of variance in plant breeding programs aiming to develop high-yielding mutant varieties. PCA and HCA are recommended algorithms for this purpose (Sudre et al., 2007). PCA is commonly used to assess the comparative contribution of variance of different variables prior to cluster analysis (Jackson, 1991). PCA enables the breeders to visualize the traits that contribute maximally toward variability. This tool also equips the breeders in selecting the best mutants for breeding purposes. In the present study, the results of PCA revealed that the first two components contributed approximately 40.4% and 33.4% of the total variability in 13 populations in the varieties Gomati VU-89 and Pusa-578, respectively. In the var. Gomati VU-89, the PC1 alone explained 28.34% of the total variation, mainly due to plant yield, harvest index, and plant height. PC2 explained 12.12% of the total variation, mainly due to pods per plant, days to maturity and days to flowering. PC1 showed positive factor loadings for all the traits. However, plant height, seed weight, and harvest index contributed negative factor loadings toward PC2. Traits such as plant yield, plant height, and harvest index were those with the highest contribution to PC1, whereas pods per plant, seeds per pod, and days to maturity were the chief contributors to PC2. In the var. Pusa-578, PC1 alone explained 21.92% of the total variation, mainly due to variations in plant yield, harvest index, and pods per plant. PC2 explained 11.55% of the total variation, mainly due to variations in days to flowering and pod length. PC1 showed positive factor loadings for all the traits. However, except for days to flowering, seeds per pod, and harvest index contributed negative factor loadings toward PC2. It is obvious that traits such as plant yield, pods per plant, pod length, and harvest index were those with the highest contribution to PC1 whereas days to maturity were the chief contributors to PC2. These results clearly indicated that PCA highlighted a few traits for exercising selection. The findings were in good agreement with the results of Raina et al. (2022) that reported that the first two PCs explained 62.4% and 17.6% of the variance and were heavily weighted by measures of plant yield and harvest index in cowpea. Further, it also revealed that first principal components contributed the highest number of traits toward genetic diversity, which may prove crucial in crop improvement programs. Days to flowering, plant height, branches per plant, pod length, and plant yield contributed to the overall variability in treated population. In breeding populations, a high level of transgressive segregation could be attained by choosing characters with high variability. The PCA biplot also provided an overview of the similarities and differences among the mutagenized populations and the interrelationships between the measured variables. The genetically similar populations, viz., 100 Gy + 0.01% SA, 200 Gy + 0.02% SA and 0.01% SA, 0.02% SA were concentrated around the origin of PC2. The genetically divergent populations, viz., 300 Gy + 0.03% SA, 400 Gy + 0.04%, and 100 Gy, 200 Gy, were placed at extreme origins in the PCA biplot. Therefore, it is important to include genetically divergent mutants in the breeding programs to broaden the genetic base.

## Conclusions

The present study confirmed that lower doses of gamma rays and sodium azide increased the agronomic traits of both cowpea varieties. Hence, for agronomic improvement of the cowpea varieties, 100 Gy, 0.01% SA, and 100 Gy + 0.01% SA are suitable for beneficial mutation inducement. Potential high-yielding putative mutants were identified in M_1_ populations that could greatly contribute to local food production and nutritional security. Based on the quantitative statistics, six higher-yielding mutagenized populations, viz., 100 Gy, 200 Gy, 0.01% SA, 0.02% SA, 100 Gy + 0.01% SA, and 200 Gy + 0.02% SA are recommended for further evaluation of high-yielding mutants. Pearson’s correlation analysis revealed that association of plant yield and seed weight was positive and highly significant, depicting that these are yield-determinative traits. Moreover, path analysis depicted that the seeds per pod followed by seed weight had the highest positive direct effect. Strong correlation and the positive direct effect of seeds per pod with plant yield and strong correlation and the negative direct effect of days to maturity with plant yield revealed that mutants with more seeds per pod and early maturing should be emphasized in the selection of high-yielding cowpea mutants. Hence, selection of cowpea mutants with a high harvest index along with concurrent consideration of short days to maturity and more seeds per pod is a prerequisite for attaining improvement in cowpea yield. HCA grouped the genetically similar and divergent populations into different clusters and confirmed mutagen-induced heterogeneous populations in the two varieties. PCA scattered the mutagenized populations over the four quadrants, with genetically similar populations concentrated around the origin and genetically divergent populations placed at extreme positions from the origin in the PCA biplot. Therefore, it is essential to include genetically divergent mutants in the breeding programs for further improvement.

## Data availability statement

The original contributions presented in the study are included in the article/**Supplementary Material**. Further inquiries can be directed to the corresponding author.

## Author contributions

AR contributed to performing the experiments, assessing data, and drafting the manuscript. SK contributed to the supervision of overall experimentation. All authors contributed to the article and approved the submitted version.
