# Supplementary material for: Field assessment of yield and its contributing traits in cowpea treated with lower, intermediate, and higher doses of gamma rays and sodium azide
Source: Front Plant Sci. 2023 Jul 14;14:1188077. doi: 10.3389/fpls.2023.1188077 (PMC10382141; doi:10.3389/fpls.2023.1188077)
Supplement: Supplementary file 1 [file DataSheet_1.docx]

***Frontiers in Plant Science***

**Crop and Product Physiology Section**

**Field assessment of yield and its contributing traits in cowpea treated with lower, intermediate, and higher doses of gamma rays and sodium azide**

*Aamir Raina^1,2*^, Samiullah Khan^1^*

^1^Mutation Breeding Laboratory, Department of Botany, Aligarh Muslim University, Aligarh, India

^2^Botany Section, Women’s College, Aligarh Muslim University, Aligarh, India

Correspondence

Dr. Aamir Raina

[aamir854@gmail.com](mailto:aamir854@gmail.com)

**Assay for nitrate reductase activity (NRA)**

Secondary leaflets of M_1_ seedlings were collected and washed thoroughly 2–3 times with running water after 10-15 days of sowing (DAS) for estimation of nitrate reductase activities (following the protocol of Jaworski, 1971). Leaves (200 mg) were cut into small pieces, chopped and collected into plastic vials. In each vial, 2.5 ml of 0.1M phosphate buffer, 0.5 ml of 0.2M potassium nitrate solution and 2.5 ml of 5% isopropanol were added (Appendix 1A-C). These vials were kept in biological oxygen demand incubator for 2 hours at 30±2 ºC in the dark. In test tubes 0.4 ml of incubated mixture is pipetted out and mixed with 0.3 ml each of sulphanilamide solution and NED-HCL (Appendix 1D-E). The test tube mixtures were left for 30 minutes for colour development and diluted to 5 ml with double-distilled water (DDW). The absorbance intensities of samples and blank (containing 4.4 ml DDW and 0.3 ml each of sulphanilamide and NED-HCL) was read at 540 nm wavelength using a spectrophotometer (Spectronic 20D, Milton Roy, USA). The absorbance intensities of each sample were compared with the calibration curve, and NRA was measured on a fresh weight basis (nmol NO_2_.g^-1^.h^-1^FW).

**Estimation of chlorophyll and carotenoid contents**

For estimation of chlorophyll and carotenoids contents, secondary leaflets of M_1_ seedlings were collected and washed thoroughly 2–3 times with running water after 10-15 DAS following the protocol of MacKinney (1941). Leaves (1g) were finely cut, ground to a fine pulp in 80% acetone (20 ml) and centrifuged at 5000 revolutions per minute (rpm) for 5 minutes (Appendix 1F). The supernatant was collected in a volumetric flask, and the residue was washed three times using 80% acetone. The absorbance intensities of samples and blank (containing 80% acetone) was read at 645 and 663 nm wavelengths for chlorophyll and 480 and 510 nm wavelengths for carotenoid contents using a spectrophotometer (Spectronic

20D, Milton Roy, USA).

**Supplementary Table 1**. Estimates of Pearson’s Correlation Coefficients between yield and yield attributing traits in varieties Gomati VU-89 and Pusa-578.

| **Pearson Correlation Coefficients, N = 390 Prob > \|r\| under H0: Rho=0** | | | | | | | | | | |
| --- | --- | --- | --- | --- | --- | --- | --- | --- | --- | --- |
| **PY** | **Var. Gomati VU-89** | | | | | | | | | |
|  | **PH** | **DF** | **DM** | **PPP** | **BPP** | **SPP** | **SW** | **PL** | **HI** |  |
|  | 0.23850 <.0001 | -0.09334 0.0656 | 0.05350 0.2919 | 0.26161 <.0001 | 0.30113 <.0001 | 0.13455 0.0078 | 0.32095 <.0001 | 0.36246 <.0001 | 0.73531 <.0001 |  |
|  | **Var. Pusa-578** | | | | | | | | | |
|  | 0.16905 0.0008 | -0.01919 0.7055 | 0.02424 0.6332 | 0.33914 <.0001 | 0.04774 0.3471 | 0.24094 <.0001 | 0.20038 <.0001 | 0.33147 <.0001 | 0.47597 <.0001 |  |

**Supplementary** **Table 2.** Characteristic means of three cluster groups of thirteen populations cowpea var. Gomati VU-89 and var. Pusa-578.

| **Characters** | **var. Gomati VU-89** | | | **var. Pusa-578** | | |
| --- | --- | --- | --- | --- | --- | --- |
|  | **Cluster 1** | **Cluster 2** | **Cluster 3** | **Cluster 1** | **Cluster 2** | **Cluster 3** |
| **Plant Height** | 182.25 | 180.14 | 175.67 | 177.76 | 180.27 | 179.25 |
| **Days to Flowering** | 79.67 | 78.97 | 78.40 | 89.00 | 87.53 | 86.43 |
| **Days to Maturity** | 150.93 | 149.47 | 148.93 | 155.90 | 156.07 | 156.80 |
| **Pods Per Plant** | 59.20 | 60.27 | 57.90 | 38.23 | 41.93 | 41.27 |
| **Branches Per Plant** | 8.73 | 9.47 | 8.53 | 11.10 | 10.60 | 11.30 |
| **Seeds Per Pod** | 11.90 | 12.40 | 12.00 | 9.37 | 10.90 | 10.00 |
| **Seed Weight** | 13.30 | 15.10 | 14.10 | 20.89 | 22.04 | 21.91 |
| **Pod Length** | 28.72 | 29.85 | 29.25 | 23.23 | 25.82 | 24.70 |
| **Plant Yield** | 93.69 | 113.02 | 97.97 | 74.70 | 100.54 | 90.36 |
| **Harvest Index** | 27.36 | 40.54 | 35.59 | 32.85 | 36.42 | 29.40 |

**Supplementary** **Table 3.** Intercluster distances among populations of cowpea var. Gomati VU-89 and var. Pusa-578.

| **Gomati VU-89** |  | CLUSTER I | CLUSTER II | CLUSTER III |  | **Pusa-578** |
| --- | --- | --- | --- | --- | --- | --- |
|  | CLUSTER I | **** | 21.21 | 10.11 | CLUSTER I |  |
|  | CLUSTER II | 19.28 | **** | 11.26 | CLUSTER II |  |
|  | CLUSTER III | 8.77 | 11.91 | **** | CLUSTER III |  |

| **var. Gomati** **VU-89** |  | **C** | **G1** | **G2** | **G3** | **G4** | **S1** | **S2** | **S3** | **S4** | **G1+S1** | **G2+S2** | **G3+S3** | **G4+S4** | **var. Pusa-578** |
| --- | --- | --- | --- | --- | --- | --- | --- | --- | --- | --- | --- | --- | --- | --- | --- |
|  | **C** |  | 19.03 | 15.09 | 8.15 | 6.54 | 22.27 | 10.82 | 9.12 | 7.56 | 17.53 | 8.40 | 6.11 | 9.15 |  |
|  | **G1** | 23.68 |  | 7.74 | 12.05 | 15.30 | 4.58 | 9.41 | 10.99 | 23.65 | 2.98 | 12.49 | 17.17 | 24.07 |  |
|  | **G2** | 19.26 | 6.09 |  | 7.68 | 11.03 | 9.64 | 8.98 | 6.76 | 18.05 | 6.30 | 7.45 | 13.62 | 19.79 |  |
|  | **G3** | 15.73 | 9.98 | 5.28 |  | 4.08 | 15.37 | 6.43 | ***2.09*** | 11.80 | 10.76 | 2.29 | 6.71 | 12.92 |  |
|  | **G4** | 11.73 | 16.72 | 12.06 | 7.08 |  | 18.82 | 8.71 | 4.88 | 9.23 | 14.03 | 4.31 | 3.80 | 9.43 |  |
|  | **S1** | 21.52 | ***3.03*** | 5.11 | 8.77 | 15.43 |  | 12.59 | 14.35 | 26.70 | 5.33 | 15.50 | 20.62 | 27.47 |  |
|  | **S2** | 19.47 | 5.09 | 3.41 | 6.48 | 13.12 | 3.31 |  | 6.11 | 16.72 | 7.93 | 6.63 | 9.26 | 16.27 |  |
|  | **S3** | 15.99 | 8.15 | 4.53 | 3.52 | 9.27 | 6.80 | 4.74 |  | 12.89 | 9.63 | 2.73 | 7.57 | 14.06 |  |
|  | **S4** | 11.69 | 15.52 | 10.47 | 6.08 | 2.68 | 14.13 | 11.91 | 8.08 |  | 22.21 | 11.73 | 9.53 | 7.63 |  |
|  | **G1+S1** | 15.49 | 9.10 | 4.23 | 4.97 | 10.42 | 7.04 | 4.77 | 3.77 | 8.66 |  | 10.76 | 15.87 | 22.84 |  |
|  | **G2+S2** | 17.06 | 8.65 | 4.99 | 5.62 | 10.75 | 7.76 | 5.66 | 3.46 | 9.46 | 4.81 |  | 6.68 | 12.85 |  |
|  | **G3+S3** | 9.07 | 20.18 | 14.88 | 11.03 | 6.09 | 18.70 | 16.25 | 12.33 | 5.69 | 12.39 | 12.70 |  | 7.17 |  |
|  | **G4+S4** | 10.01 | 26.88 | 21.88 | 17.29 | 10.53 | 25.34 | 22.93 | 18.97 | 11.66 | 19.36 | 19.77 | 7.76 |  |  |

**Supplementary** **Table 4.** Interpopulation proximity matrix based on Euclidean distance in the different populations of cowpea var. Gomati VU-89 and var. Pusa-578.

C = Control; G1 = 100 Gy γ rays; G2 = 200 Gy γ rays; G3 = 300 Gy γ rays; G4 = 400 Gy γ rays; S1 = 0.01% SA; S2 = 0.02% SA; S3 = 0.03% SA; S4 = 0.04% SA; G1 +S1 = 100 Gy γ rays+0.01% SA; G2+S2 = 200 Gy γ rays+0.02% SA; G3+S3 = 300 Gy γ rays+0.03% SA; G4+S4 = 400 Gy γ rays+0.04% SA.

**Supplementary** **Table 5.** Principal components analysis showing the contribution of ten quantitative traits among populations of cowpea var. Gomati VU-89 and var. Pusa-578.

| **Characters** | **PRINCIPAL COMPONENTS (PCS)** | | | |
| --- | --- | --- | --- | --- |
|  | **var. Gomati VU-89** | | **var. Pusa-578** | |
|  | PC 1 | PC 2 | PC 1 | PC 2 |
| **Plant Height** | 0.436 | -0.208 | 0.197 | -0.210 |
| **Days to Flowering** | 0.106 | 0.344 | 0.026 | 0.666 |
| **Days to Maturity** | 0.093 | 0.425 | 0.101 | -0.226 |
| **Pods Per Plant** | 0.199 | 0.554 | 0.375 | -0.181 |
| **Branches Per Plant** | 0.269 | 0.111 | 0.166 | -0.019 |
| **Seeds Per Pod** | 0.102 | 0.331 | 0.297 | 0.161 |
| **Seed Weight** | 0.265 | *-0.345* | 0.184 | *-0.545* |
| **Pod Length** | 0.293 | 0.243 | 0.371 | 0.212 |
| **Plant Yield** | **0.525** | -0.078 | **0.532** | -0.042 |
| **Harvest Index** | 0.483 | -0.204 | 0.486 | 0.236 |
| **EIGEN VALUE** | 2.834 | 1.212 | 2.192 | 1.155 |
| **% OF VARIANCE** | 28.349 | 12.123 | 21.921 | 11.558 |
| **% CUMMULATIVE VARIANCE** | 28.349 | 40.472 | 21.921 | 33.479 |

**Supplementary Figure 1:** Structure of mutagen Sodium Azide.


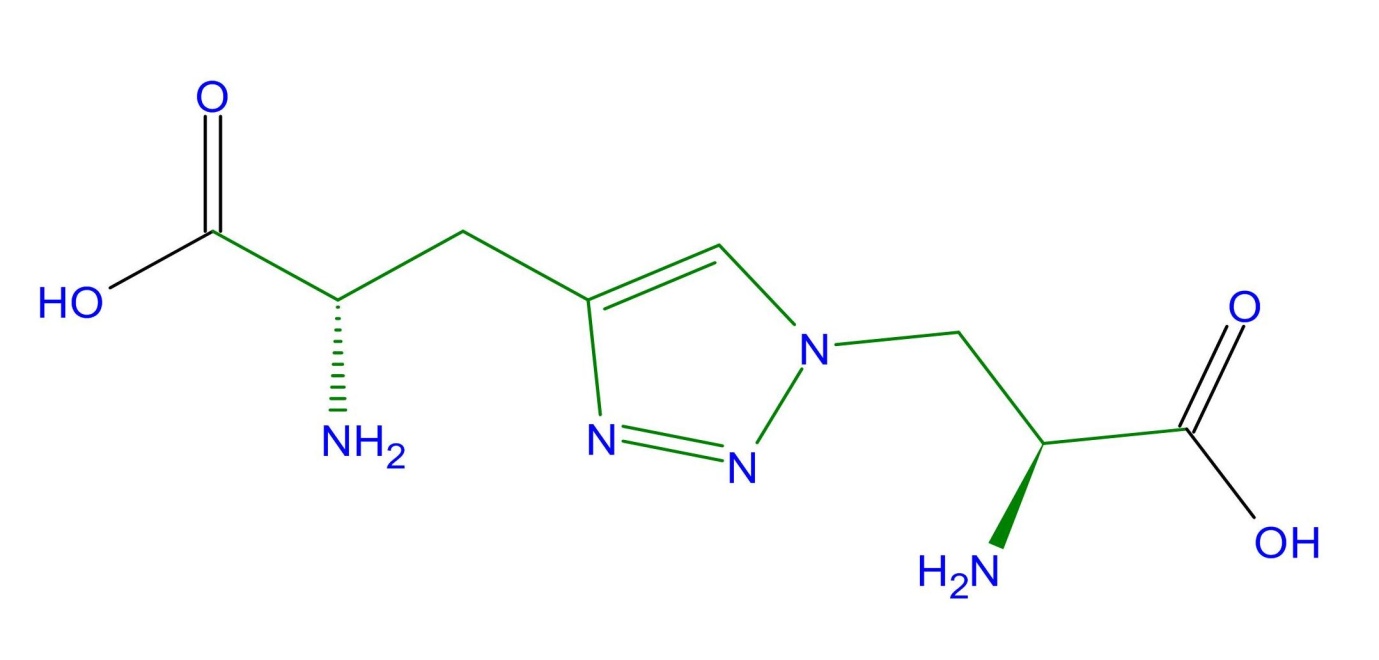


**Supplementary Fig. 2** Field plots layout of M_1_ generations in a randomized complete block design (RCBD) for each cultivar (Source: Raina et al., 2020. http://creativecommons.org/licenses/by/4.0/).


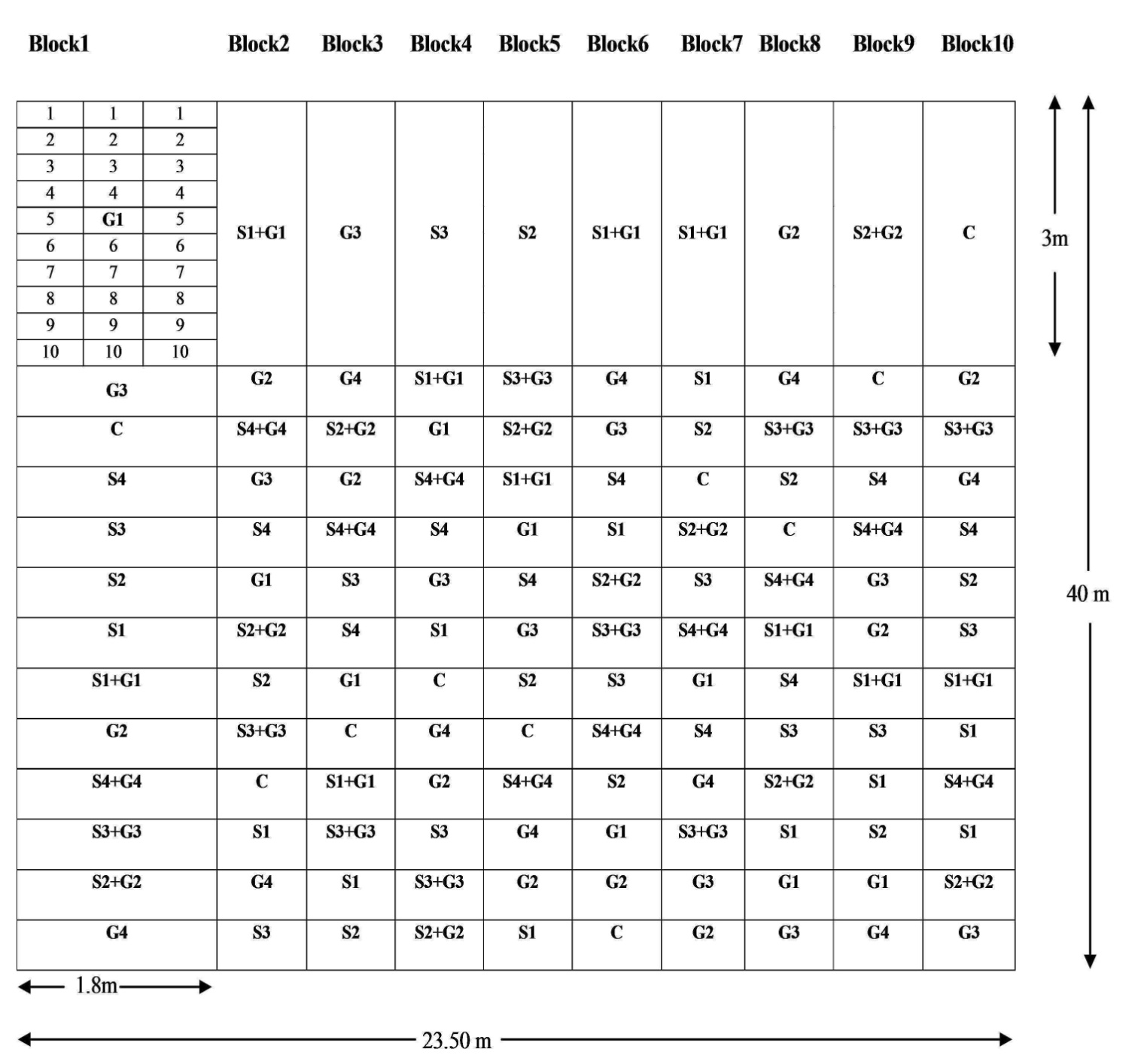


**Appendix I: Reagents used for the estimation of NRA, chlorophyll and carotenoid contents.**

**(A) 0.1M phosphate buffer (7.4 pH)**

27.2 g of KH2PO4 and 45.63 g of K2HPO4.7H2O were dissolved separately in 1000 ml of DDW.

The above solution of KH2PO4 and K2HPO4.7H2O were mixed in the ratio of 36:64, respectively.

**(B) 0.2M potassium nitrate**

20.2 g of KNO3 was dissolved in sufficient DDW and final volume was made upto 1000 ml using DDW

**(C) Isopropanol (5%)**

5 ml of isopropanol was pipette into sufficient DDW and final volume was made upto 100 ml, using DDW.

**(D) Sulphanilamide (1%)**

1 g of sulphamnilamide was dissolved in 100 ml of 3N HCL.

3N HCL was prepared by dissolving 25.86 ml of HCl in sufficient DDW and final volume was maintained to 100 ml, using DDW.

**(E) N-1-nehthyl-ethylenediamine dihydro chloride-HCl (NED-HCl) (0.02%)**

20 mg of NED-HCl was dissolved in sufficient DDW and final volume was made upto 100 ml, using DDW.

**(F) Acetone (80%)**

80% acetone was prepared bu mixing 80 ml of acetone with 20 ml of DDW.

**(G) Acid mixture**

The acid mixture was made by nitric, sulfuric, and perchloric acids in the ratio 10: 1: 4 by volume, respectively.
